# Bioactive constituents from the edible seaweed *Halymenia hawaiiana* (Rhodophyta)

**DOI:** 10.1080/13880209.2025.2521285

**Published:** 2025-06-26

**Authors:** Achara Raksat, Md Samiul Huq Atanu, Karla J. McDermid, Marisa M. Wall, Boon Loong Chang, Supakit Wongwiwatthananukit, Leng Chee Chang

**Affiliations:** ^a^ Department of Pharmaceutical Sciences, The Daniel K. Inouye College of Pharmacy, University of Hawaiʻi at Hilo, Hilo, Hawaiʻi, USA; ^b^ Department of Marine Science, University of Hawaiʻi at Hilo, Hilo, Hawaiʻi, USA; ^c^ Daniel K. Inouye U.S. Pacific Basin Agricultural Research Center, Hilo, Hawaiʻi, USA; ^d^ Department of Traditional Chinese Medicine, School of Pharmacy, Management and Science University, Shah Alam, Selangor, Malaysia; ^e^ Department of Pharmacy Practice, The Daniel K. Inouye College of Pharmacy, University of Hawaiʻi at Hilo, Hilo, Hawaiʻi, USA

**Keywords:** Marine macroalga, Halymenia hawaiiana, anti-microbial, anti-inflammatory, cytotoxicity

## Abstract

**Context:**

The marine macroalga, *Halymenia hawaiiana*, holds significant commercial potential due to its culinary uses among various ethnic groups in Hawaiʻi and its success in aquaculture.

**Objective:**

To investigate the chemical components and potential medicinal properties of *H. hawaiiana*.

**Materials and methods:**

Dried, ground *H. hawaiiana* was sequentially extracted with three solvents: ethyl acetate, methanol, and *n*-butanol. Chromatographic procedures were sequentially applied, leading to the isolation of several compounds. Structure determination of these compounds was performed using spectroscopic methods. The isolated compounds were then assessed through several *in vitro* assays.

**Results:**

A total of 11 compounds were isolated and identified: two nucleosides (**1** and **2**), a cytosine analog (**3**), five saturated long-chain fatty acids (**4**–**8**), cholesterol (**9**), and two of its derivatives (**10**–**11**) were isolated. Compounds **10** and **11** displayed promising antimicrobial activity against *Staphylococcus aureus*, both exhibiting MIC values of 8 μg/mL, and methicillin-susceptible *S. aureus*, with MIC values of 32 and 64 μg/mL, respectively. Furthermore, using a cell culture model, compounds **9**, **10**, and **11** exhibited significant anti-inflammatory effects as indicated by their inhibition of lipopolysaccharide (LPS)-induced nitric oxide (NO) production, with IC_50_ values ranging from 50.2 to 60.8 µM. These compounds also effectively inhibited the generation of reactive oxygen species (ROS)/reactive nitrogen species (RNS) in RAW 264.7 mouse macrophage cells with IC_50_ values of 55–73.7 µM without inducing cytotoxicity. Compounds **9–11** also exhibited mild cytotoxicity against the non-small cell lung cancer cell line A549, with compound **10** eliciting the strongest response (IC_50_ 86.1 µM).

**Conclusions:**

This study uncovered a diverse array of constituents from *H. hawaiiana*. Notably, compounds **10** and **11**, which feature peroxide side chains, show significant promise as lead compounds for the development of novel anti-inflammatory and antimicrobial agents.

## Introduction

The demand for natural products (NPs) is increasing because of their potential therapeutic properties and positive effects on human health. Approximately 40% of drugs approved by the FDA between 1981 and 2014 have their origins in NPs, indicating a growing trend (Newman and Cragg 2012).

Marine macroalgae or seaweeds are emerging as a promising source of NPs, with a history of use in various cultures worldwide, including Pacific Islander, Asian, European, Native American, and South American populations (Dillehay et al. 2008; Baghel 2023; Baghel et al. 2023). The rising popularity of seaweed products is likely because of their potential to provide essential micronutrients and bioactive phytochemicals (Ho and Redan 2022). Roughly 1,800 brown (class Phaeophyceae), 6,200 red (phylum Rhodophyta), and 1,800 green (phylum Chlorophyta) macroalgal species are found in the marine environment (Pereira 2021). The Hawaiian Islands have a rich marine flora with 658 seaweeds: 71 brown algae, 450 red algae, and 137 green algae (Sherwood and Guiry 2023).

One genus of marine red algae, *Halymenia*, is found extensively in tropical regions throughout the Indo-Pacific area. A common species, *Halymenia durvillei Bory,* is used as a food source and a natural coloring agent (Vivithanaporn et al. 2023). In the Hawaiian Islands, a related native species, *Halymenia hawaiiana* J.J. Hernández-Kantún & A.R. Sherwood, previously erroneously identified as *H. formosa* (Abbott 1999; Hernández-Kantún et al. 2012) is found in the shallow subtidal zone. The reddish-brown to purplish-red, foliose thallus grows in a flattened, fan-like blade with one to three orders of tapering branches and can reach lengths of up to 30 cm. The thallus surface is covered with small soft spines. In Hawaiʻi, *H. hawaiiana* is used as food, and is a valuable source of protein, vitamins, minerals, and dietary fiber (McDermid and Stuercke 2003; McDermid et al. 2005, 2019).

There have been reports highlighting the anticancer, antioxidant, and antimicrobial activities of *H. durvillei* (Sanger et al. 2019; Arguelles 2022; Kasmiati et al. 2022; Sangpairoj et al. 2022; Khatulistiani et al. 2023) found in the Indo-Pacific region. However, those studies primarily focus on biological activity extracts and do not provide much information on their chemical compositions. A study by Tassakka et al. (2021) reported the analysis of 37 compounds using gas chromatography and mass spectrometry (GC-MS). The ethanol extract contains caryophyllene, eucalyptol, and 1,4,7-cycloundecatriene, whereas 1,5,9,9-tetramethyl-, Z, Z, Z- were reported in the ethyl acetate (EtOAc) extraction. The identified compounds primarily consist of fatty acids or lipids derived from this seaweed. The EtOAc extracts from *Halymenia porphyroides* Bϕrgesen were analyzed and found to contain several major compounds, including fatty acids such as hexadecanoic acid and its methyl ester, as well as cholesterol. Additionally, 7-oxocholesterol was identified as a minor compound in *H. porphyroides.* The structures of these compounds were determined using low-resolution proton nuclear magnetic resonance (NMR) and gas chromatography mass spectrometry (GC-MS) spectroscopy (Bano et al. 1987).

In our ongoing search for bioactive compounds derived from nature, *H. hawaiiana* was selected for further study because of its commercial potential, culinary applications in Hawaiʻi, and its success in aquaculture. This investigation presents the isolation and characterization of eleven compounds derived from *H. hawaiiana*. These include two nucleosides (**1** and **2**), a cytosine analog (**3**), five saturated long-chain fatty acids (**4**–**8**), and three cholesterol derivatives (**9**–**11**). The structures of these compounds were determined using spectroscopic data analysis. The cytotoxicity in the A549 cells, LPS-induced nitric oxide and ROS/RNS inhibitory effect in Raw 264.7 cells, antioxidant and antimicrobial activities of these isolated compounds were also investigated.

## Materials and methods

### General experimental procedures

One-dimensional- and two-dimensional NMR spectra, were recorded on a Bruker AVANCE DRX-400 NMR spectrometer (Bruker, Billerica, MA, USA) at 400 MHz for ^1^H and 100 MHz for ^13^C. The spectra were processed using MestReNova version 14.2.1-27684 software, with CDCl_3_ (*δ*_H_ 7.23, *δ*_C_ 77.16) or CD_3_OD (*δ*_H_ 3.31, *δ*_C_ 49.0) as solvents. Sephadex LH-20 (GE Healthcare, Piscataway, NJ, USA) and Silica gel (230–400 mesh, 480–800 mesh, Sorbent Technologies, Atlanta, GA, USA) were used for column chromatography (CC). Thin-layer chromatography was performed on precoated silica gel F_254_ 0.25 mm glass-backed plates (Sorbent Technologies, Atlanta, GA, USA).

### Culture, extraction and isolation

*Halymenia hawaiiana*, originally collected from a wild population on Hawaiʻi Island, was grown outdoors in 560 L flat-bottom and 400 L conical tanks at the University of Hawaiʻi Hilo Pacific Aquaculture and Coastal Resources Center (PACRC) in Hilo, HI. Tanks were supplied with aeration, flowing seawater, pumped from a 140 m deep seawater well onshore at the PACRC with a temperature of 20-23 °C. The fresh *H. hawaiiana* was harvested on June 11, 2022. Voucher specimens (BISH 798487) were deposited at the Bernice Pauahi Bishop Herbarium. Identification was performed by K. J. McDermid (mcdermid@hawaii.edu) following current references (Abbott 1999; Hernández-Kantún et al. 2012). The fresh *H. hawaiiana* (3.4 kg) was washed with fresh water to remove surface salts, and epiphytes, then placed in a lyophilizer for sample drying. The dried *H. hawaiiana* (284.6 g) was ground into a fine powder and extracted with organic solvents: ethyl acetate (3 L, EtOAc), methanol (3 L, MeOH), and *n*-butanol (3 L, BuOH) three times for each at room temperature and concentrated under reduced pressure to give EtOAc (916.2 mg), MeOH (21.7 g), and BuOH (3.4 g) partitions. The ethyl acetate-soluble partition (916.2 mg) was separated over a CC on Sephadex LH-20 using 5% dichloromethane (CH_2_Cl_2_) in MeOH as eluent to afford three subfractions (E_A_−E_C_). Subfraction E_B_ (814.3 mg) was subjected to Si gel CC eluted with CH_2_Cl_2_−MeOH (98:2, v/v) in gradients, to yield compounds **4** (4.6 mg), **5** (5.3 mg), **6** (11.9 mg), **7** (64.7 mg), and **8** (1.1 mg). The MeOH partition (21.7 g) was separated over CC on Sephadex LH-20 using 100% MeOH followed by reversed-phase C_18_ using MeOH − H_2_O (6:4, v/v) to give **9** (2.3 mg), **10** (7.8 mg) and **11** (2.7 mg). The BuOH partition (3.4 g) was separated by CC over silica gel using acetone − hexanes (3:7, v/v) followed by CC using MeOH − CH_2_Cl_2_ (1:9, v/v) to yield **1** (3.5 mg), **2** (8.3 mg), and **3** (0.7 mg).

### Sulforhodamine B (SRB) cytotoxicity assay

A549 human lung carcinoma epithelial cells (CCL-185^TM^) and NIH/3T3 murine fibroblast (CRL-1658 ^™^) were obtained from the American Type Culture Collection (ATCC, Manassas, VA, USA). Cells were cultured in Dulbecco’s modified Eagle’s medium (DMEM, Cat. No. 12800-082, Invitrogen, Massachusetts, USA) supplemented with 10% fetal bovine serum (FBS, Cat. No. 26140, Gibco BRL Co, Grand Island, NY, USA), and antibiotics (penicillin G and streptomycin, Cat. No. P4458, Roche, Sigma-Aldrich, USA) at 37 °C in a humidified 5% CO_2_ incubator. Cytotoxic activity was assessed using the sulforhodamine B (SRB) assay (Cat. No. S9012, Roche, Sigma-Aldrich, USA) as described by Islam et al. (2023). A549 cells were seeded in 96-well plates (Cat. No. 10861-666, VWR Chemicals, USA) at a density of 6,000 cells/well and incubated for 24 h. Subsequently, cells were treated with increasing concentrations of the sample (1.25–100 μg/mL) or 0.5% dimethyl sulfoxide (DMSO, Cat. No. D8418, Sigma-Aldrich, USA) (control) for 72 h. Cisplatin (Cat. No. 232120, Sigma-Aldrich, USA) (0.5–20 μM) served as a positive control. After incubation, cells were fixed with trichloroacetic acid (Cat. No. T4885, Sigma-Aldrich, USA), stained with SRB (Cat. No. S9012, Sigma-Aldrich, USA), and the absorbance was measured at 515 nm. Percent cell death was calculated using the following equation:

$$\text{\% Cell death}=\frac{\left(\text{ODc}-\text{OD}0)‐(\text{ODs}-\text{OD}0\right)}{\left(\text{ODc}-\text{OD}0\right)}\times 100$$

OD_0_ = Optical density of cells before treatment (baseline reading).

OD_C_ = Optical density of untreated control cells after 72 h.

OD_S_ = Optical density of cells treated with the test sample after 72 h.

The median inhibitory concentration (IC_50_) at 50% cell death was determined by plotting % cell death against test sample concentration. Non-linear regression analysis was performed using GraphPad Prism to calculate the IC_50_ value. This value reflects the concentration needed to inhibit cell growth by 50%.

#### Inhibition of nitric oxide (NO) generation in lipopolysaccharide-induced murine macrophage RAW 264.7 cells

The nitrite concentration in the culture medium was measured to indicate nitric oxide (NO) production as previously described (Min et al. 2009). The murine macrophage RAW 264.7 cell line was obtained from ATCC, Manassas, VA, USA, and maintained in Dulbecco’s modified Eagle’s medium supplemented with 10% heat-inactivated FBS (Cat. No. A5670801, Gibco BRL Co, Grand Island, NY, USA), penicillin G (100 units/mL) (Cat. No. P7794, Sigma-Aldrich, USA), streptomycin (100 µg/mL) (Cat. No. S9137, Sigma-Aldrich, USA), and L-glutamine (2 mM) (Cat. No. 25030081, Gibco BRL Co, Grand Island, NY, USA). In brief, RAW 264.7 cells were seeded at a density of 4 × 10^4^ cells/well in phenol red-free media (Cat. No. 11835030, Gibco, USA) in a 96-well plate, and incubated in a humidified incubator with 5% CO_2_ at 37 °C for 24 hr. Following this, the cells were treated with increasing concentrations of samples ranging from 10–50 μg/mL for 4 hr, after which the cells were treated with 1 μg/mL LPS for 20 h. L-N-Monomethyl Arginine citrate (L-NMMA) (Cat. No. NC0190932, Cayman Chemical, Michigan, USA), a non-selective inhibitor of NOS, was used as positive control. The concentration of nitric oxide production was assessed using the Griess reagent, which consists of a 1:1 (v/v) mixture of 1% sulfanilamide (Cat No. A13001, Alfa Aesar, Massachusetts, USA) in H_3_PO_4_ and *N*-(1-naphthyl)ethylenediamine dihydrochloride (Cat. No. 102397, MP Biomedicals, USA). Absorbance was recorded with a microplate reader (SynergyMx, BioTeK, Winooski, VT, USA) at 540 nm. A standard curve was created using sodium nitrite (1–100 μM) (Cat. No. 44227, Alfa Aesar, Massachusetts, USA). A sulforhodamine B (SRB) assay was employed to assess the cellular viability of the samples with RAW 264.7 cells under a similar experimental setup. The final % inhibition of NO production was adjusted based on cell viability.

#### Measurement of reactive oxygen species/reactive nitrogen species (ROS/RNS) levels in raw 264.7 cells

To measure the formation of intracellular ROS and RNS, we utilized the fluorogenic dye 2′,7′-dichlorodihydrofluorescein diacetate (Cat. No. 4091-99-0, H_2_DCF-DA, Abcam, Boston, MA, USA). This dye detects hydrogen peroxide, peroxyl radicals, and peroxynitrite anions (Kwon et al. 2019). In brief, RAW 264.7 cells were seeded into a black 96-well plate (Cat. No. 165305, Thermo Fisher, USA) at 4 × 10^4^ cells per well and incubated for 24 hr at 37 °C in 5% CO_2_. Afterward, the cells were treated with or without a sample for 4 hr before adding 1 μg/mL LPS (Cat. No. 00-4976-93, Thermo Fisher, USA) for 20 hr. Once this incubation was complete, the cells were washed twice with fetal bovine serum (FBS)-free media (Cat. No. A5256701, Thermo Fisher, USA) and stained with 10 μM H2DCF-DA (Cat. No. D399, Thermo Fisher, USA) in FBS-free media for 30 min at 37 °C, protected from light. Next, the dye-containing media was discarded, and the cells were washed twice with fresh serum-free media. The relative fluorescent intensity of the cells was measured using a fluorescence plate reader (Synergy H1, BioTeK, Winooski, VT, USA) with excitation/emission at 485 nm/535 nm. Curcumin (Cat. No. C1386-5G, Millipore Sigma, Burlington, MA, USA), a natural phenolic compound with antioxidant activity, was used as positive control. All samples were tested in triplicates. A sulforhodamine B (SRB) assay was conducted to assess cellular viability under a similar experimental setup. The final percentage of inhibition of ROS/RNS generation was calibrated using the % of cell viability.

#### Ferric-reducing antioxidant power (FRAP) assay

The FRAP assay was used to evaluate antioxidant activity based on the ability of the sample to reduce Fe^3+^-TPTZ (2,4,6-tris(2-pyridyl)-*S*-triazine) to Fe^2+^-TPTZ (Benzie and Strain 1996). This reduction results in a color change from colorless to blue. The absorbance was measured at 593 nm using a Microplate Reader (BioTek ELx800 Instruments Inc. Winooski, Vermont, USA) with the Gen 5 software for FRAP value calculations. A ferrous sulfate heptahydrate (FeSO_4_·7H_2_O) (Cat. No. 215422, Sigma-Aldrich, USA) standard curve, ranging from 1000 µM to 50 µM, was prepared for the assay. The FRAP reagent consists of 300 mM sodium acetate buffer (pH 3.6) (Cat. No. 90358, Sigma-Aldrich, USA), 10 mM TPTZ solution (Cat. No. T1253, Sigma-Aldrich, USA), and 20 mM ferric (III) chloride hexahydrate (FeCl_3_·6H_2_O) (Cat. No. 236489, Sigma-Aldrich, USA) in a 10:1:1 ratio, respectively, and heated to 37 °C for ten minutes before use. A blank was measured at 593 nm with 150 µL FRAP reagent added to each well of a 96-well microtiter plate. Samples (20 µL of each sample) (1, 0.5, 0.25 mg/mL) and ascorbic acid (Sigma-Aldrich, Cat. No. A4403) positive control (1 mg/mL) were added in triplicates. Reducing capacity was expressed by FRAP values in an µM FeSO_4_/µg sample unit.

#### Antibacterial assay

##### Bacteria strains

Methicillin-resistant *Staphylococcus aureus* (MRSA), methicillin-susceptible *S. aureus* (MSSA), and Gram-negative clinical isolates of bacteria [*Escherichia coli* (9637) and *Serratia marcescens* ATCC 13880] were used for this study. MRSA USA-300 LAC, *S. aureus* Newman, *S. aureus* 8325-4, *E. coli*, and *S. marcescens* were obtained from the American Type Culture Collection (ATCC, Manassas, Virginia, USA). MSSA HBP10, MSSA LUU7, MSSA R113, *S. aureus* ONE9, and MSSA POB2 were isolated in Hawaiʻi (Gerken et al. 2021). Mueller Hinton Broth (MHB, GTIN:00382902979630, Becton, Dickinson and Company, Franklin Lakes, NJ, USA) and Mueller Hinton Agar (MHA, GTIN:00382902252504, Becton, Dickinson and Company, Franklin Lakes, NJ, USA,) were used for bacterial inoculation. MRSA was a pathogen strain causing hospital-acquired infections in 1982. This strain easily acquires resistance to antibiotics, such as vancomycin. MSSA strains isolated from the Island of Hawaiʻi included: Honolʻii Beach Park (HBP10) carry the genes erm(C), aph(3′)-III, and mecA, which provide resistance to macrolide, aminoglycoside, and beta-lactam antibiotics, respectively; Wailuku River (LUU7) which contains the blaZ gene (beta-lactam resistance) (Gerken et al. 2021). Richardson’s Beach Park (R113) and Onekahakaha (ONE9) which is known for its virulence factors (hlgA, hlgB, hlgC, lukD, and lukE).

The minimum inhibitory concentrations (MICs) were determined using a two-fold serial dilution method with MHB following the recommendations of the Clinical and Laboratory Standards Institute (CLSI 2012; Elshikh et al. 2016). In brief, serial two-fold dilutions of the samples in DMSO were mixed with MHB in a 96-well microplate. Then, 50 µL of bacteria suspension was added to each well, resulting in a final concentration of 1 × 10^4^ colony-forming units/well. The plates were incubated at 35–37 °C for 18–24 hr. After incubation, 10 µL of 0.18% resazurin (Cat. No. R7017, Sigma-Aldrich, USA) was added to each well (Elshikh et al. 2016). The MIC was assessed within 2–3 hr after the addition of resazurin. All antimicrobial assays were performed in triplicate, using vancomycin (Cat. No. SBR00001, Sigma-Aldrich, USA) and gentamicin (Cat. No. G1397, Sigma-Aldrich, USA) as standard controls.

##### Statistical analysis

All data are presented as mean ± standard error of the mean (SEM) and were collected from three separate experiments. Statistical analyses were conducted using Student’s *t*-test or ANOVA, followed by Tukey’s multiple comparisons test. Significance is indicated by *p < 0.05, **p < 0.01, and ***p < 0.001. The IC_50_ values of A549 cells were calculated through non-linear curve fit analysis using Prism software (GraphPad 10.0.2, San Diego, USA), with *R*^2^ > 0.9 and *p* > 0.5 (runs test) as parameters of goodness of fit.

## Results and discussion

**Figure 1.**
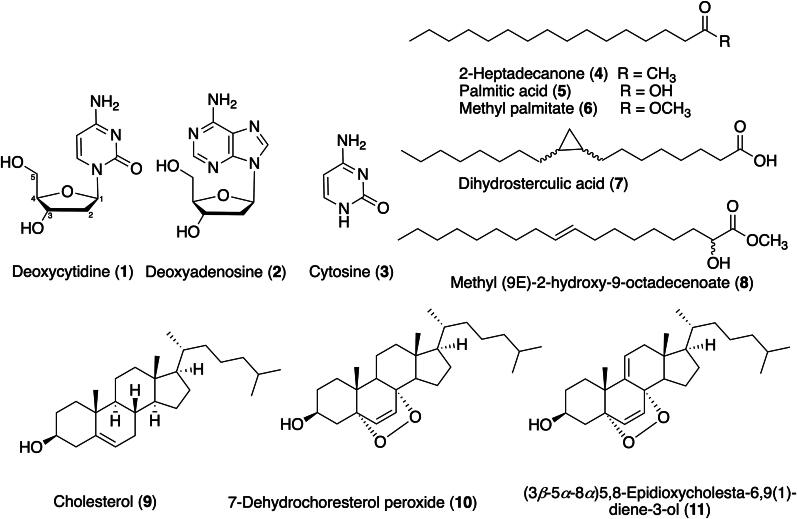
Structures of compounds **1–11** from *H. hawaiiana* extract.

We identified eleven compounds from *H. hawaiiana*: two nucleosides; deoxycytidine (**1**) (Jessop et al. 2009) and deoxyadenosine (**2**) (Watson et al. 1965), a cytosine analog; cytosine (**3**) (Barker and Marsh 1964), five saturated long-chain fatty acids; 2-heptadecanone (**4**) (Pino et al. 2005), palmitic acid (**5**) (Sadrolhosseini et al. 2017), methyl palmitate (**6**) (Lee et al. 2010), dihydrosterculic acid (**7**) (Knothe 2006), methyl (9*E*)-2-hydroxy-9-octadecenoate (**8**) (Ross et al. 1946; Hwang and Erhan 2001), cholesterol (**9**) (Bano et al. 1987; Minn et al. 2004), and two cholesterol derivatives, 7-dehydrochoresterol peroxide (**10**) (Minn et al. 2004), (3*β*-5*α*-8*α*)5,8-epidioxycholesta-6,9(1)-diene-3-ol (**11**) (Guyot and Durgeat 1981) (Figure 1). The structures of the isolated compounds were determined based on their NMR spectroscopic data and from comparisons with those previously reported.

This is the first study to focus on these compounds in H. hawaiiana. A closely related species, H. durvillei, also has interesting natural products. For example, extracts contain steroids, flavonoids, triterpenoids, saponins, and hydroquinones, many of which are bioactive and may have pharmaceutical applications (Sanger et al. 2019; Tassakka et al. 2021; Arguelles 2022). Another Halymenia sp., collected from the coast of Madagascar, was extracted using ethyl acetate and methanol. Interestingly, cholesterol was identified as the major sterol component (Rahelivao et al. 2015). Brown algae (phaeophyta) predominantly contain phytosterols and a small proportion of plant cholesterol. In contrast, red algae (Rhodophyta) contain cholesterol as their principal sterol content, with a minor quantity of phytosterols such as fucosterol (Sohn et al. 2021).

In the current study, compounds **1**, **2**, and **3** were obtained as white solids. The NMR data for compound **1** revealed the presence of a pyrimidine-type cytosine linked to a ribose sugar that lacks a hydroxyl group at the C-2 position. Similarly, compound **2** features a purine-type adenine connected to a ribose that also lacks a hydroxyl group at the same position as in compound **1**. Therefore, structures **1** and **2** have nucleoside skeletons and were identified as deoxycytidine (Jessop et al. 2009) and deoxyadenosine (Watson et al. 1965), respectively. Analysis of the ^1^H NMR data of compound **3** revealed it is an analog of compound **1**. However, the ribose sugar in compound **1** was absent. A comparison of the NMR data of compound **3** with those of cytosine (Barker and Marsh 1964) confirmed its structure.

Compounds **4**–**8** were obtained as white powders. The NMR data of compounds **4**–**8** indicated that they were long-chain fatty acids. Compound **4** displayed two methyls, fourteen methylenes, and one carbonyl carbon. The structure of compound **4** was confirmed by comparison of its NMR spectroscopic data with the literature values and was identified as 2-heptadecanone (Pino et al. 2005). The NMR data of compounds **5** and **6** suggested that their structures were closely related to compound **4**, except that the methyl ketone of **4** was replaced by a hydroxy in compound **5** and methoxy in compound **6**. Thus, compounds **5** and **6** were established as palmitic acid (which is also known as hexadecanoic acid) (Sadrolhosseini et al. 2017) and methyl palmitate, respectively (Lee et al. 2010). The data obtained with compound **7** were nearly identical to those of palmitic acid (**5**) except for the signals of a tricyclic ring. Compound **7** was then characterized as dihydrosterculic acid (Knothe 2006). The NMR spectroscopic data of compound **8** was very close to those of palmitic acid (**6**) except that compound **8** displayed resonances for two *trans* olefinic protons and an oxy-methine proton. Thus, compound **8** was identified as methyl (9*E*)-2-hydroxy-9-octadecenoate by comparison of its spectra with those reported in the literature (Ross et al. 1946; Hwang and Erhan 2001).

Compounds **9**, **10**, and **11** were isolated as light yellow gums. Analysis of the NMR data (^1^H proton and ^13^C carbon) of compound **9** revealed the presence of 27 sterol-like carbons, including three quaternary, eight methines, eleven methylenes, and five methyl carbons. Analysis of the NMR data of compounds **10** and **11** also revealed that they are structurally related to compound **9**. However, compounds **10** and **11** displayed two oxygen-bearing quaternary carbons. Therefore, **9**, **10**, and **11** were identified as cholesterol (Minn et al. 2004), 7-dehydrochoresterol peroxide (3*β*-5*α*-8*α*) (Minn et al. 2004), and 5,8-epidioxycholesta-6,9(1)-diene-3-ol (Guyot and Durgeat 1981), respectively. While compounds **10** and **11** have been previously isolated from various marine organisms, including tunicates (Abourriche et al. 2000), long-spined sea urchins (Minn et al. 2004), marine sponges (Gauvin et al. 2000; Jeong et al. 2019), and sea hares (Miyamoto et al. 1988), this study marks the first identification of these compounds from a seaweed source.

Regarding biological activity, the ethanolic and ethyl acetate extracts of *H. durvillei* were evaluated for activity against SARS-CoV-2 using a computational approach, focusing on their inhibitory effects on the main protease (3CL-Mpro) (Tassakka et al. 2021). Approximately 37 compounds were extracted and analyzed using GC-MS. Among them, two compounds, 1-2 tetradecandiol, and E, E, Z-1,3,12-nonadecatriene-5,14-diol, were identified as potential therapeutic agents. These compounds demonstrated competitive affinity scores against the 3 chymotrypsin-like protease Main Protease (3CL-Mpro), suggesting they may have potential activity against COVID-19 (Tassakka et al. 2021). 3CL-Mpro is fundamental for the replication of SARS-CoV-2.

Additionally, Sangpairoj et al. (2022) reported that the hexane solvent fraction of *H. durvillei* primarily contains hexadecanoic acid, also known as palmitic acid, which was isolated from *H. hawaiiana* in this study. Hexadecanoic acid promotes apoptosis and autophagy in cancer cell lines, specifically in human triple-negative breast cancer cells (MDA-MB-231 cells). The extract of hexadecanoic acid induces cytotoxic effects through mitochondrial membrane dysfunction, DNA damage, and endoplasmic reticulum stress (Sangpairoj et al. 2022). In addition, the anticancer effects of an ethyl acetate extract derived from *H. durvillei* on HT-29 colorectal cancer cells have been reported (Chantree et al. 2023). These effects were confirmed through the induction of apoptosis, autophagy, and cell cycle arrest, all facilitated by the regulation of the phosphoinositide 3-kinase (PI3K)/protein kinase B (AKT)/mammalian target of rapamycin (mTOR) signaling pathway.

**Figure 2.**
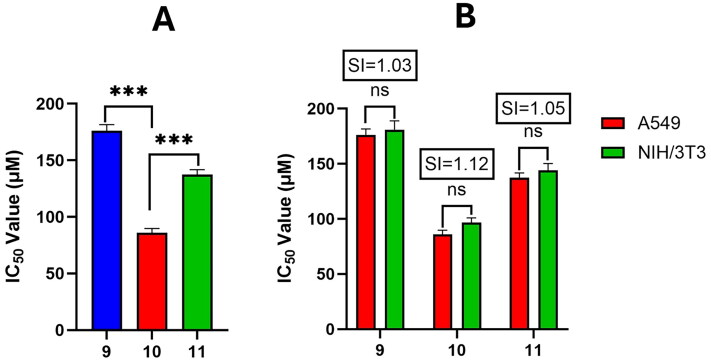
**(A)** The cytotoxic effects of cholesterol (**9**) and its derivatives (**10** and **11**) on A549 cells were determined using the SRB assay after 72 hrs of exposure. (B) a comparison of the cytotoxicity of compounds **9**, **10**, and **11** on A549 (cancerous) and NIH/3T3 (non-cancerous) cell lines was conducted, with the respective selectivity index (SI) measured by SRB assay after 72 h. The results are presented as mean values ± SD (*n* = 3). Statistical significance is indicated by *p < 0.05, **p < 0.01, and ***p < 0.001.

In the present study, we investigated the effects of various compounds on non-small cell lung cancer cells (A549). Cholesterol (**9**) and its derivatives **10**, and **11** demonstrated mild cytotoxicity, with IC_50_ values of 176.3 ± 5.3, 86.1 ± 3.6, and 137.4 ± 4.4 μM, respectively (Figure 2(A)). In contrast, nucleosides (**1** and **2**), cytosine (**3**), and saturated long-chain fatty acids (**4**–**8**) showed no cytotoxic response, even at the highest concentration (100 µg/ml) tested. Despite prior evidence of the cytotoxicity of compound **5** (palmitic acid, also known as hexadecanoic acid), it was inactive in terms of cytotoxicity towards A549 cell line, likely due to its poor solubility in cell culture media. Although compound **10** exhibited activity significantly higher than compounds (**9** and **11**), all were less effective than a positive control chemotherapeutic drug, cisplatin (IC_50_ 4.2 ± 0.3 μM). The cytotoxicity of compounds **9**, **10**, and **11** was analyzed on A549 lung cancer cells in comparison to non-tumorigenic NIH/3T3 cells. The IC_50_ values in NIH/3T3 were 180.9 ± 8.04, 96.58 ± 4.34 and 144.15 ± 6.15 μM, respectively. The Selectivity Index values (SI) for these compounds were 1.03, 1.12 and 1.05 respectively (Figure 2(B)), indicating that these compounds did not demonstrate significant selectivity towards cancer cells.

Nevertheless, Tian et al. (2017) reported anti-cancer activity in 7-dehydrocholesterol peroxide (compound **10** which we isolated from H. hawaiiana) along with its acetate and hemisuccinate derivatives (Tian et al. 2017). These cholesterol peroxide derivatives demonstrated improved anticancer activity and selectivity compared to ergosterol peroxide, indicating their potential as new chemotherapeutic agents (Tian et al. 2017). Ergosterol peroxide itself is a promising natural product with anticancer properties against several cancer cell lines (Ling et al. 2024).

Nitric oxide (NO) is a reactive nitrogen species produced from L-arginine by nitric oxide synthase (NOS). NO plays a crucial role in normal physiological functions such as neurotransmission, vasodilation, and immune defense. Among the isoforms of NOS, the inducible NOS (iNOS) is consistently associated with chronic inflammation (Korhonen et al. 2005; Nomelini et al. 2008). Additionally, excessive NO formation promotes the inflammatory response and increases oxidative stress and tissue damage (Saini and Singh 2019). Lipopolysaccharide (LPS), a natural stimulator of immune and inflammatory responses, activates signaling pathways in RAW 264.7 cells which in turn leads to the release of cytokines, nitric oxide (NO), and prostaglandin E2 (PGE2) (Liu et al. 2022). To assess the pro-inflammatory response of LPS in RAW 264.7 cells, the amount of nitrite, a stable NO product, was measured in the cell culture medium. Although 7-dehydrocholesterol (7-DHC) was not isolated in this study, 7-DHC was reported to be synthesized and produced by some marine algae. 7-DHC was susceptible to free radical autoxidation, which led to the formation of oxysterols, including 7-dehydrocholesterol peroxide was reported (Korade et al. 2010). Interestingly, our study isolated 7-dehydrocholesterol peroxide (5α,8α-epidioxycholest-6-ene-3β-ol, compound **10**) from *H. hawaiiana* (Karode et al. 2010).

**Figure 3.**
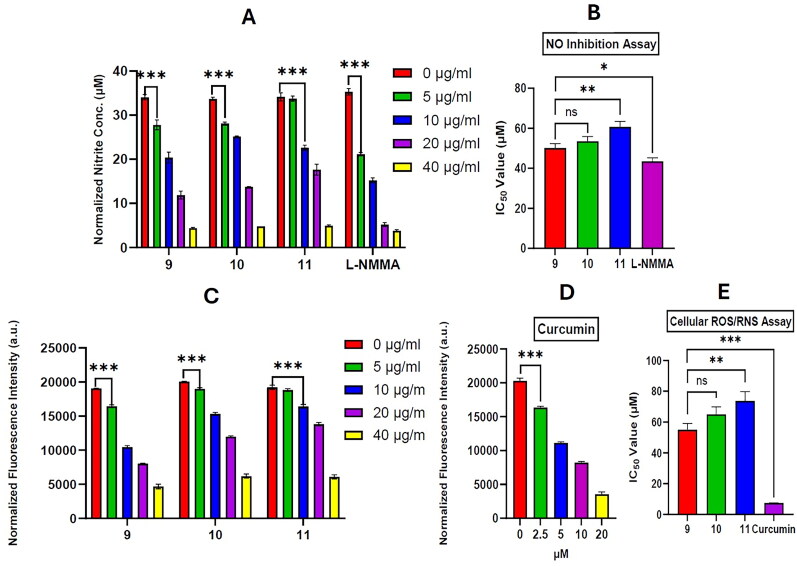
(A) The inhibition of NO production by cholesterol (**9**), its derivatives (**10** and **11**), and L-NMMA was evaluated in LPS (1 μg/mL)-induced RAW 264.7 cells at various concentrations (5-40 µg/ml). (B) The IC_50_ values of compounds **9**–**11**, and L-NMMA in the NO inhibition assay conducted in LPS-induced RAW264.7 cells. (C) The suppression of intracellular reactive oxygen species (ROS) and reactive nitrogen species (RNS) production by compounds **9**, **10**, **11** (5–40 µg/ml), and (D) Curcumin (2.5–20 µM) was evaluated using the H_2_DCFDA fluorogenic dye in LPS-induced RAW 264.7 cells. (E) The IC_50_ values for compounds **9**, **10**, **11**, and curcumin in the intracellular ROS/RNS assay, measured using the H2DCFDA dye in LPS-induced RAW264.7 cells, were calculated. The results are expressed as mean values ± SD (*n* = 3). Statistical significance is indicated by *p < 0.05, **p < 0.01 and ***p < 0.001.

In this study, cholesterol (**9**) and its derivatives **10**, and **11** showed a response indicative of anti-inflammatory activity by lowering the NO production in a concentration-dependent manner (5–40 µg/ml) with compounds **9** and **10** exhibiting significant (*p* > 0.001) inhibition at 5 µg/ml (Figure 3(A)). These compounds exhibited IC_50_ values of 50.2 ± 2.2, 53.6 ± 2.3 and 60. 8 ± 2.7 µM, respectively (Figure 3(B)). Both compounds **9** and **10** showed significantly (*p* < 0.01) higher activity than compound **11**. Whereas, nucleosides (**1** and **2**), cytosine (**3**), and saturated long-chain fatty acids (**4**–**8**) showed no significant NO inhibitory activity, even at the highest concentration (50 μg/mL). L-NMMA (positive control) exhibited significantly (*p* < 0.05) higher NO suppressing effect compared to compound **9** with IC_50_ value of 43.5 ± 1.8 µM.

In addition to NO, a wide variety of free radicals, i.e., hydroxyl ions (HO^−^), superoxide (O_2_^•−),^ etc., are generated in response to LPS. Prior studies have demonstrated that excessive ROS production leading to oxidative stress plays a major role in the inflammatory processes and results in tissue injury (Seo et al. 2015). To assess the ROS/RNS formation with RAW264.7 cells, a fluorogenic dye, 2′,7′-dichlorodihydrofluorescein diacetate (H_2_DCF-DA), was utilized. Incorporation of H_2_DCF-DA into cells leads to its conversion to 2′,7′-dichlorodihydrofluorescein (H_2_DCF) by the action of intracellular esterases, which in turn oxidized to fluorescent DCF by ROS/RNS (López et al. 2006). Here, cholesterol (**9**) and derivatives (**10** and **11**) demonstrated inhibition of LPS-induced ROS/RNS generation in a concentration-dependent manner (IC_50_ 55 ± 4.1, 65 ± 4.8 and 73.7 ± 6.2 µM, respectively) (Figure 3(C,E)). Again, compounds **9** and **10** demonstrated significantly higher oxidative stress suppression compared to compound **11** (*p* < 0.01). Curcumin, the standard antioxidant, demonstrated a concentration-dependent suppression of ROS and RNS in the range of 2.5–20 µM. It also exhibited significantly (*p* < 0.001) lower IC_50_ value (7.6 ± 0.1 µM) compared to compounds **9**, **10** and **11** (see Figure 3(D,E)).

Seaweeds are rich in phenolic compounds, flavonoids, and phlorotannins that possess significant antioxidant and antibacterial properties, making them relevant for pharmaceutical applications. Antioxidant properties of the water extract from *H. durvillei* were demonstrated. The extract contained a high content of R-Phycoerythrin along with strong antioxidant activity. Furthermore, microencapsulation was proposed as an effective method for preserving the antioxidant activity of the water extract (Khatulistiani et al. 2023). Additional work established a total phenolic content (TPC) of 6.77 ± 0.03 mg Gallic Acid Equivalent (GAE) per gram of *H. durvillei* Borry 1828 extract. Further, *H. durvillei* exhibited potent ABTS^+^ [2,2′-azino-bis (3-ethylbenzothiazoline-6-sulfonic acid)] radical scavenger capability and high copper reduction capacity, with IC_50_ values of 106 µg GAE/mL and 20.44 µg/GAE/mL, respectively (Arguelles 2022). These findings underscore the potential of *H. durvillei* to combat oxidative stress and enhance health due to bioactive components.

In the current study, the three partitions of *H. hawaiana* crude extract (EtOAc, MeOH, and BuOH) and five isolated compounds (**1**, **5**–**7**, and **10**) showed weak antioxidant activity as measured by their FRAP values (0.07–6.24 µM/µg; Table 1). For comparison, the positive controls, ascorbic acid, and green tea, yielded FRAP values of 35.59 and 38.59 µM/µg, respectively.

**Table 1.** Antioxidant activity (FRAP) of crude extracts and isolation compounds.

Samples	Absorbance	FRAP (µM)	FRAP (µM/µg)
*H. hawaiiana* (EtOAc)	0.11	164.73	2.06 ± 0.02
*H. hawaiiana* (MeOH)	0.06	80.73	1.01 ± 0.01
*H. hawaiiana* (BuOH)	0.03	5.40	0.07 ± 0.01
**1**	0.01	−32.60	0.41 ± 0.03
**5**	0.07	88.06	1.10 ± 0.02
**6**	0.27	499.40	6.24 ± 0.07
**7**	0.14	230.06	2.88 ± 0.03
**10**	0.13	204.07	2.55 ± 0.02
Ascorbic acid	1.45	2847.40	35.59 ± 0.09
Green tea	1.61	3167.40	39.59 ± 0.11

*R^2^= 0.9954*.

The antimicrobial and toxicity of methanol and hexane extracts of *H. durvillei* red seaweed have been reported by Kasmiati et al. (2022). The bacterial strains tested were *E. coli*, *Salmonella typhi*, *Pseudomonas aeruginos*a, *Aeromonas hydrophila*, and *Vibrio harveyi* at a dose of 200 µg/disk. Extract toxicity was tested on *Artemia salina* larvae at concentrations in the range of 1,000–31.25 µg/mL in two-fold serial dilutions. The results indicated that the methanol and hexane extracts had the highest activity against *S. typhi* and *A. hydrophila*, with zones of inhibition (ZOI) of 26.2 and 21 mm, respectively. The IC_50_ inhibitory concentration was 98.24 µg/mL for methanol extract against *S. typhi* (Kasmiati et al. 2022).

In the current study, three crude extracts of H. hawaiana (EtOAc, MeOH, and BuOH) and isolated compounds were tested for antimicrobial activity against eight Gram-positive (MSSA and MRSA) and two Gram-negative *(*E. coli and Serratia marcescens*)* bacteria as presented in Table 2. The MeOH extract showed moderate antimicrobial activity against *S. aureus* NEWMAN, with an MIC of 320 μg/mL. This aligns with the results for cholesterol derivatives **10** and **11**, which were isolated from the MeOH crude extract and also exhibited notable antimicrobial activity against *S. aureus* NEWMAN, with both having MICs of 8 μg/mL. Both of these derivatives (**10** and **11**) also demonstrated moderate antimicrobial activities against methicillin-susceptible S. aureus (MSSA) (HBP10) with MIC values of 32 and 64 μg/mL, respectively. HBP10 is a strain of MSSA isolated from Hawaiʻi that carries the genes erm(C), aph(3′)-III, and mecA, which provide resistance to macrolide, aminoglycoside, and beta-lactam antibiotics, respectively (Gerken et al. 2021). MSSA LUU7 is a strain that contains the *blaZ* gene (beta-lactam resistance); whereas ONE6 is known for its virulence factors (*hlgA*, *hlgB*, *hlgC*, l*ukD*, and *lukE*). Two nucleosides (**1**–**2**) and a cytosine analog (**3**) showed MIC values in the range of 64–128 μg/mL. In contrast, fatty acid derivatives (**4**–**8**) did not exhibit any activity against Gram-positive or Gram-negative bacterial strains. In a related species, *H. dilata Zanardini*, the sulfated galatan component demonstrated similar effects against bacteria, as well as anticancer and antioxidant activity (Vinosha et al. 2024).

**Table 2.** MIC Values (µg/mL) of compounds from *H. hawaiiana* extract.

Compounds	Gram-positive									Gram-negative	
	MRSA POB2	MRSA USA300	MRSA HBP10	MSSA R113	MSSA LUU7	*S. aureus* ONE9	*S. aureus* CART817	*S. aureus* 8325-4	*S. aureus*NEWMAN	*E. coli*	*S. marcescent*
*H. hawaiiana (EtOAc)*	–	Inactive	–	1280	Inactive	1280	1280	Inactive	Inactive	Inactive	1280
*H. hawaiiana (MeOH)*	–	1280	–	1280	1280	1280	1280	1280	320	1280	1280
*H. hawaiiana (BuOH)*	–	1280	–	1280	1280	1280	1280	1280	1280	1280	1280
**1**	128	128	128	128	128	128	128	128	128	128	128
**2**	128	128	128	128	128	128	128	128	128	128	64
**3**	128	128	128	128	128	128	128	128	128	128	128
**4**	Inactive	Inactive	Inactive	Inactive	Inactive	Inactive	Inactive	Inactive	Inactive	Inactive	Inactive
**5**	Inactive	Inactive	Inactive	Inactive	Inactive	Inactive	Inactive	Inactive	Inactive	Inactive	Inactive
**6**	Inactive	Inactive	Inactive	Inactive	Inactive	Inactive	Inactive	Inactive	Inactive	Inactive	Inactive
**7**	Inactive	Inactive	Inactive	Inactive	Inactive	Inactive	Inactive	Inactive	Inactive	Inactive	Inactive
**8**	Inactive	Inactive	Inactive	Inactive	Inactive	Inactive	Inactive	Inactive	Inactive	Inactive	Inactive
**9**	128	128	128	128	128	128	128	128	64	128	128
**10**	128	128	32	128	128	128	128	128	8	128	64
**11**	128	128	64	128	128	128	128	128	8	128	64
Vancomycin	0.5	0.5	1	0.5	0.5	0.5	1	0.5	0.5	–	–
Gentamicin	–	–	–	–	–	–	–	–	–	0.25	0.25

*Inactive indicated a MIC value >128 µg/mL.

Our results indicate that compounds **9**, **10**, and **11** exhibited anti-inflammatory effects by the inhibition of lipopolysaccharide (LPS)-induced nitric oxide (NO) production, with IC_50_ values of 50.2–60.8 µM. These compounds also inhibited the generation of reactive oxygen species (ROS)/reactive nitrogen species (RNS) in RAW 264.7 mouse macrophage cells with IC_50_ values of 55–73.7 µM without cytotoxicity. Lipopolysaccharide (LPS) is mainly located in the outer cell wall of Gram-negative bacteria (Noailles et al. 2018). Compounds **9**–**11** inhibited LPS-induced nitric oxide production in RAW 264 cells, which may be part of their antibacterial mechanism of action. In addition, artemisinin is known for its endoperoxide bridge (C-O-O-C), which may drive its potent antimalarial and/or biological activity. The mode of action: the endoperoxide bridge is cleaved by heme or molecular iron, resulting in the production of free radicals and alkylating intermediates. These then target specific malaria membrane-associated proteins, causing significant damage (Meshnick 1994; Meshnick et al. 1996; Dhingra et al. 2000; Wang et al. 2021). Similarly, compounds **10** and **11** also contain endoperoxide bridges (C-O-O-C) and could operate *via* a similar mechanism to deliver their antibacterial effects.

## Conclusions

The current study uncovered a variety of secondary metabolites from the edible Hawaiian marine macroalga *H. hawaiiana*. These metabolites include nucleosides, cytosine analogs, fatty acids, cholesterol and its derivatives. Notably, two cholesterol derivatives (**10** and **11**) were identified for the first time from a seaweed source. Both compounds demonstrated anti-inflammatory activity, although they also exhibited mild cytotoxic effects. These compounds also exhibited significant antimicrobial properties against *S. aureus* Newman. These cholesterol derivatives **10** and **11** feature peroxide side chains and show promise as lead compounds for development as antimicrobial agents. These data provide objective evidence for the potential value of these edible seaweeds. In particular, as a food, the anti-inflammatory activity of *H. hawaiiana* could prove beneficial. In addition, the substance could prove to be a promising source for skincare products targeting bacterial skin conditions, given its demonstrated activity against *S. aureus*.

## Supplementary Material

4 SI R2.pdf

## Data Availability

The datasets used and/or analyzed are available from the corresponding author upon reasonable request.

## References

[CIT0001] Abbott IA. 1999. Marine red algae of the Hawaiian Islands. Honolulu, Hawaiʻi: Bishop Museum Press; p. 477.

[CIT0002] Abourriche A, Charrouf M, Chaib N, Bennamara A, Bontemps N, Francisco C. 2000. Isolation and bioactivities of epidioxysterol from the tunicate *Cynthia savignyi*. Farmaco. 55(6–7):492–494. doi: 10.1016/s0014-827x(00)00040-9. 11204751

[CIT0003] Arguelles ED. 2022. Bioactive properties of *Halymenia durvillei* Bory 1828 for pharmaceutical application: antioxidant, antidiabetic, antiwrinkling, and skin-whitening activities. Yuzuncu Yil Univ J Agric Sci. 32(1):57–68. doi: 10.29133/yyutbd.1016050.

[CIT0004] Baghel RS. 2023. Developments in seaweed biorefinery research: a comprehensive review. J Chem Eng. 454:140177. doi: 10.1016/j.cej.2022.140177.

[CIT0005] Baghel RS, Choudhary B, Pandey S, Pathak PK, Patel MK, Mishra A. 2023. Rehashing our insight of seaweeds as a potential source of foods, nutraceuticals, and pharmaceuticals. Foods. 12(19):3642. doi: 10.3390/foods12193642. 37835294 PMC10573080

[CIT0006] Bano S, Shafiuddin Perveen S, Bano N, Ahmad VD, Shameel M. 1987. Chemical constituents of red algae *Acanthophora dendroides* and *Halymenia porphyroides*. Pak J Bot. 19(2):253–257.

[CIT0007] Barker DL, Marsh RE. 1964. The crystal structure of cytosine. Acta Cryst. 17(12):1581–1587. doi: 10.1107/S0365110X64003899.

[CIT0008] Benzie IF, Strain JJ. 1996. The ferric reducing ability of plasma (FRAP) as a measure of “antioxidant power”: the FRAP assay. Anal Biochem. 239(1):70–76. doi: 10.1006/abio.1996.0292. 8660627

[CIT0009] Chantree P, Martviset P, Sornchuer P, Thongsepee N, Sangpairoj K, Meemon K, Niamnont N, Tamtin M, Sobhon P. 2023. Ethyl acetate extract of *Halymenia durvillei* induced apoptosis, autophagy, and cell cycle arrest in colorectal cancer cells. Prev Nutr Food Sci. 28(1):69–78. doi: 10.3746/pnf.2023.28.1.69. 37066031 PMC10103606

[CIT0010] CLSI. 2012. Reference method for dilution antimicrobial susceptibility test for bacteria that grow aerobically; approved standard M07-A9. Wayne (PA): Clinical and Laboratory Standards Institute.

[CIT0011] Dhingra V, Pakki SR, Narasu ML. 2000. Antimicrobial activity of artemisinin and its precursors. Curr. Sci. 78:709–713.

[CIT0012] Dillehay TD, Ramírez C, Pino M, Collins MB, Rossen J, Pino-Navarro JD. 2008. Monte verde: seaweed, food, medicine, and the peopling of South America. Science. 320(5877):784–786. doi: 10.1126/science.1156533. 18467586

[CIT0013] Elshikh M, Ahmed S, Funston S, Dunlop P, McGaw M, Marchant R, Banat IM. 2016. Resazurin-based 96-well plate microdilution method for the determination of the minimum inhibitory concentration of biosurfactants. Biotechnol Lett. 38(6):1015–1019. doi: 10.1007/s10529-016-2079-2. 26969604 PMC4853446

[CIT0014] Gauvin A, Smadja J, Aknin M, Faure R, Gaydou EM. 2000. Isolation of bioactive 5α, 8α-epidioxy sterols from the marine sponge *Luffariella* cf. *variabilis*. Can J Chem. 78(7):986–992.

[CIT0015] Gerken TJ, Roberts MC, Dykema P, Melly G, Lucas D, De Los Santos V, Gonzalez J, Butaye P, Wiegner TN. 2021. Environmental surveillance and characterization of antibiotic-resistant *Staphylococcus aureus* at coastal beaches and rivers on the island of Hawaiʻi. Antibiotics. 10(8):980. doi: 10.3390/antibiotics10080980. 34439030 PMC8388868

[CIT0016] Guyot M, Durgeat M. 1981. Occurrence of 9 (11)-unsaturated sterol peroxides in tunicates. Tetrahedron Lett. 22(15):1391–1392. doi: 10.1016/S0040-4039(01)90329-6.

[CIT0017] Hwang HS, Erhan SZ. 2001. Highly selective asymmetric synthesis of 2‐hydroxy fatty acid methyl esters through chiral oxazolidinone carboximides. J Amer Oil Chem Soc. 78(2):205–211. doi: 10.1007/s11746-001-0244-9.

[CIT0018] Hernández-Kantún JJ, Sherwood AR, Riosmena-Rodriguez R, Huisman JM, De Clerck O. 2012. Branched *Halymenia* species (Halymeniaceae, Rhodophyta) in the Indo-Pacific region, including descriptions of *Halymenia hawaiiana sp. nov*. and *H. tondoana sp. nov*. Eur J Phycol. 47(4):421–432. doi: 10.1080/09670262.2012.733734.

[CIT0019] Ho KK, Redan BW. 2022. Impact of thermal processing on the nutrients, phytochemicals, and metal contaminants in edible algae. Crit Rev Food Sci Nutr. 62(2):508–526. doi: 10.1080/10408398.2020.1821598. 32962399 PMC9109159

[CIT0020] Islam MA, Atanu MSH, Siraj MA, Acharyya RN, Ahmed KS, Dev S, Uddin SJ, Das AK. 2023. Supplementation of syringic acid-rich *Phrynium pubinerve* leaves imparts protection against allergic inflammatory responses by downregulating iNOS, COX-2, and NF-κB expressions. Heliyon. 9(2):e13343. doi: 10.1016/j.heliyon.2023.e13343. 36816283 PMC9932742

[CIT0021] Jeong H, Latif A, Kong CS, Seo Y, Lee YJ, Dalal SR, Cassera MB, Kingston DG. 2019. Isolation and characterization of antiplasmodial constituents from the marine sponge *Coscinoderma* sp. Z Naturforsch C J Biosci. 74(11–12):313–318. doi: 10.1515/znc-2019-0039. 31393837

[CIT0022] Jessop TC, Tarver JE, Carlsen M, Xu A, Healy JP, Heim-Riether A, Fu Q, Taylor JA, Augeri DJ, Shen M, et al. 2009. Lead optimization and structure-based design of potent and bioavailable deoxycytidine kinase inhibitors. Bioorg Med Chem Lett. 19(23):6784–6787. doi: 10.1016/j.bmcl.2009.09.081. 19836232

[CIT0023] Kasmiati K, Nurunnisa AT, Amran A, Resya MI, Rahmi MH. 2022. Antibacterial activity and toxicity of *Halymenia durvillei* red seaweed from Kayangan island, South Sulawesi, Indonesia. Fish Aquat Sci. 25(8):417–428. doi: 10.47853/FAS.2022.e38.

[CIT0024] Khatulistiani TS, Oktavia DA, Munifah I, Marraskuranto E. 2023. Antioxidant and anti-tyrosinase activities of *Halymenia durvillei* water extract containing R-phycoerythrin before and after microencapsulation. Squalen Bull Mar Fish. 18(1):21–930.

[CIT0025] Knothe G. 2006. NMR characterization of dihydrosterculic acid and its methyl ester. Lipids. 41(4):393–396. doi: 10.1007/s11745-006-5110-x. 16808153

[CIT0026] Korade Z, Xu L, Shelton R, Porter NA. 2010. Biological activitives of 7-dehydrocholesterol-derived oxysterols: implications for Smith-Lemli-Opitz syndrome. J Lipid Res. 51(11):3259–3269. doi: 10.1194/jlr.M009365. 20702862 PMC2952566

[CIT0027] Korhonen R, Lahti A, Kankaanranta H, Moilanen E. 2005. Nitric oxide production and signaling in inflammation. Curr Drug Targets Inflamm Allergy. 4(4):471–479. doi: 10.2174/1568010054526359. 16101524

[CIT0028] Kwon DH, Cha HJ, Lee H, Hong SH, Park C, Park SH, Kim GY, Kim S, Kim HS, Hwang HJ, et al. 2019. Protective effect of glutathione against oxidative stress-induced cytotoxicity in RAW 264.7 macrophages through activating the nuclear factor erythroid 2-related factor-2/heme oxygenase-1 pathway. Antioxidants. 8(4):82. doi: 10.3390/antiox8040082. 30939721 PMC6523540

[CIT0029] Lee YC, Chang HH, Liu CH, Chen MF, Chen PY, Kuo JS, Lee TJF. 2010. Methyl palmitate: a potent vasodilator released in the retina. Invest Ophthalmol Vis Sci. 51(9):4746–4753. doi: 10.1167/iovs.09-5132. 20357193

[CIT0030] Ling T, Arroyo-Cruz LV, Smither WR, Seighman EK, Martínez-Montemayor MM, Rivas F. 2024. Early preclinical studies of ergosterol peroxide and biological evaluation of its derivatives. ACS Omega. 9(35):37117–37127. doi: 10.1021/acsomega.4c04350. 39246459 PMC11375702

[CIT0031] Liu Y, Chen W, Zheng F, Yu H, Wei K. 2022. Xanthatin alleviates LPS-induced inflammatory response in RAW264. 7 macrophages by inhibiting NF-κB, MAPK and STATs activation. Molecules. 27(14):4603. doi: 10.3390/molecules27144603. 35889477 PMC9322085

[CIT0032] López E, Arce C, Oset-Gasque MJ, Cañadas S, González MP. 2006. Cadmium induces reactive oxygen species generation and lipid peroxidation in cortical neurons in culture. Free Radic Biol Med. 40(6):940–951. doi: 10.1016/j.freeradbiomed.2005.10.062. 16540389

[CIT0033] McDermid KJ, Stuercke B. 2003. Nutritional composition of edible Hawaiian seaweeds. J Appl Phycol. 15(6):513–524. doi: 10.1023/B:JAPH.0000004345.31686.7f.

[CIT0034] McDermid KJ, Stuercke B, Haleakala OJ. 2005. Total dietary fiber content in Hawaiian marine algae. Bot Mar. 48:437–440.

[CIT0035] McDermid KJ, Martin KJ, Haws MC. 2019. Seaweed resources of the Hawaiian Islands. Bot Mar. 62(5):443–462. doi: 10.1515/bot-2018-0091.

[CIT0036] Meshnick SR. 1994. The mode of action of antimalarial endoperoxides. Trans R Soc Trop Med Hyg. 88 Suppl 1(1):S31–S32. doi: 10.1016/0035-9203(94)90468-5. 8053021

[CIT0037] Meshnick SR, Taylor TE, Kamchonwongpaisan S. 1996. Artemisinin and the antimicrobial endoperoxides: from herbal remedy to targeted chemotherapy. Microbiol Rev. 60(2):301–315. doi: 10.1128/mr.60.2.301-315.1996. 8801435 PMC239445

[CIT0038] Minn CV, Kiem PV, Huong LM, Kim YH. 2004. Cytotoxic constituents of *Diadema setosum*. Arch Pharmacal Res. 27:734–737. 10.1007/BF02980141 15357000

[CIT0039] Min H-Y, Kim MS, Jang DS, Park E-J, Seo E-K, Lee SK. 2009. Suppression of lipopolysaccharide-stimulated inducible nitric oxide synthase (iNOS) expression by a novel humulene derivative in macrophage cells. Int Immunopharmacol. 9(7–8):844–849. Epub 2009 Mar 17. doi: 10.1016/j.intimp.2009.03.005. 19298870

[CIT0040] Miyamoto T, Honda M, Sugiyama S, Higuchi R, Komori T. 1988. Studies on the constituents of marine opisthobranchia, III. Isolation and structure of two 5, 8α‐epidioxysterols and a cholesteryl ester mixture from the albumen gland of *Aplysia juliana*. Liebigs Ann Chem. 1988(6):589–592. doi: 10.1002/jlac.198819880616.

[CIT0041] Newman DJ, Cragg GM. 2012. Natural products as sources of new drugs over the 30 years from 1981 to 2010. J Nat Prod. 75(3):311–335. doi: 10.1021/np200906s. 22316239 PMC3721181

[CIT0042] Noailles A, Maneu V, Campello L, Lax P, Cuenca N. 2018. Systemic inflammation induced by lipopolysaccharide aggravates inherited retinal dystrophy. Cell Death Dis. 9(3):350. doi: 10.1038/s41419-018-0355-x. 29500424 PMC5834451

[CIT0043] Nomelini RS, Ribeiro LCA, Murta BMT, Adad SJ, Murta EFC. 2008. Production of nitric oxide and expression of inducible nitric oxide synthase. Mediators Inflamm. 2008:186584. doi: 10.1155/2008/186584. 19132106 PMC2613969

[CIT0044] Pereira L. 2021. Macroalgae. Encyclopedia. 1(1):177–188. doi: 10.3390/encyclopedia1010017.

[CIT0045] Pino JA, Mesa J, Muñoz Y, Martí MP, Marbot R. 2005. Volatile components from mango (*Mangifera indica* L.) cultivars. J Agric Food Chem. 53(6):2213–2223. doi: 10.1021/jf0402633. 15769159

[CIT0046] Rahelivao MP, Gruner M, Andriamanantoanina H, Andriamihaja B, Bauer I, Knölker H-J. 2015. Red algae (Rhodophyta) from the coast of Madagascar: preliminary bioactivity studies and isolation of natural products. Mar Drugs. 13(7):4197–4216. doi: 10.3390/md13074197. 26198236 PMC4515612

[CIT0047] Ross J, Gebhart AI, Gerecht JF. 1946. The addition of olefinic esters to maleic anhydride. J Am Chem Soc. 68(7):1373–1376. doi: 10.1021/ja01211a084.

[CIT0048] Sadrolhosseini AR, Abdul Rashid S, Zakaria A. 2017. Synthesis of gold nanoparticles dispersed in palm oil using laser ablation technique. J Nanomater. 2017:1–5. doi: 10.1155/2017/6496390.

[CIT0049] Saini R, Singh S. 2019. Inducible nitric oxide synthase: an asset to neutrophils. J Leukoc Biol. 105(1):49–61. doi: 10.1002/JLB.4RU0418-161R. 30285282

[CIT0050] Sanger G, Rarung LK, Damongilala LJ, Kaseger BE, Montolalu LADY. 2019. Phytochemical constituents and antidiabetic activity of edible marine red seaweed (*Halymenia durvilae*). IOP Conf Ser Earth Environ Sci. 278(1):012069. doi: 10.1088/1755-1315/278/1/012069.

[CIT0051] Sangpairoj K, Settacomkul R, Siangcham T, Meemon K, Niamnont N, Sornkaew N, Tamtin M, Sobhon P, Vivithanaporn P. 2022. Hexadecanoic acid-enriched extract of *Halymenia durvillei* induces apoptotic and autophagic death of human triple-negative breast cancer cells by upregulating ER stress. Asian Pac J Trop Biomed. 12(3):132–140. doi: 10.4103/2221-1691.338922.

[CIT0052] Seo DW, Yi YJ, Lee MS, Yun BS, Lee SM. 2015. Differential modulation of lipopolysaccharide-induced inflammatory cytokine production by and antioxidant activity of fomentariol in RAW264. 7 cells. Mycobiology. 43(4):450–457. doi: 10.5941/MYCO.2015.43.4.450. 26839505 PMC4731650

[CIT7585673] Sherwood AR, Guiry MD. 2023. Inventory of the seaweeds and seagrasses of the hawaiianislands. Biology. 12(2):215. doi: 10.3390/biology12020215. 36829491 PMC9953416

[CIT0053] Sohn S-I, Rathinapriya P, Balaji S, Jaya Balan D, Swetha TK, Durgadevi R, Alagulakshmi S, Singaraj P, Pandian S. 2021. Phytosterols in seaweeds: an overview on biosynthesis to biomedical applications. Int J Mol Sci. 22(23):12691. doi: 10.3390/ijms222312691. 34884496 PMC8657749

[CIT0054] Tassakka AC ,Sumule O ,Massi MN ,Manggau M ,Iskandar IW ,Alam JF ,Permana AD ,Liao LM. 2021. Potential bioactive compounds as SARS-CoV-2 inhibitors from extracts of the marine red alga *Halymenia durvillei* (Rhodophyta) − A computational study. Arab J Chem. 14(11): 103393–103407. doi: 10.1016/j.arabjc.2021.103393. 34909061 PMC8381616

[CIT0055] Tian N-n, Li C, Tian N, Zhou Q-x, Hou Y-J, Zhang B-w, Wang X-s 2017. Synthesis of 7-dehydrocholesterol peroxides and their improved anticancer activity and selectivity over ergosterol peroxide. New J Chem. 41(24):14843–14846. doi: 10.1039/C7NJ04100D.

[CIT0056] Vivithanaporn P, Siangcham T, Tanawoot V, Settacomkul R, Pranweerapaiboon K, Meemon K, Niamnont N, Tamtin M, Sobhon P, Sangpairoj K. 2023. Apoptotic and autophagic cell death effects of the hexane extract of tropical marine algae *Halymenia durvillei* against human glioblastoma cells: in vitro and in silico studies. Trends Sci. 21(2):7157. doi: 10.48048/tis.2024.7157.

[CIT0057] Vinosha M, Palanisamy S, Jeneeta S, Rajasekar P, Marudhupandi T, Karthikeyan M, Mohandoss S, You S, Prabhu NM. 2024. Antibacterial, anticancer and antioxidant effects of sulfated galactan from *Halymenia dilata*: in vitro and in vivo analysis. Food Biosci. 60:104420. doi: 10.1016/j.fbio.2024.104420.

[CIT0058] Wang X, Bittner T, Milanov M, Kaul L, Mundinger S, Koch HG, Jessen-Trefzer C, Jessen HJ. 2021. Pyridinium modified anthracenes and their endoperoxides provide a tunable scaffold with activity against gram-positive and gram-negative bacteria. ACS Infect Dis. 7(8):2073–2080. doi: 10.1021/acsinfecdis.1c00263. 34291902

[CIT0059] Watson DG, Sutor DT, Tollin P. 1965. The crystal structure of deoxyadenosine monohydrate. Acta Crystallogr. 19(1):111–124. doi: 10.1107/s0365110x65002852. 5896865

