# Supplementary material for: Bioactive constituents from the edible seaweed Halymenia hawaiiana (Rhodophyta)
Source: Pharm Biol. 2025 Jun 26;63(1):447–59. doi: 10.1080/13880209.2025.2521285 (PMC12203697; doi:10.1080/13880209.2025.2521285)
Supplement: 4 SI R2.pdf [file IPHB_A_2521285_SM1538.pdf]

SUPPLEMENTARY MATERIALS

Bioactive constituents from the edible seaweed *Halymenia hawaiiana* (Rhodophyta)

Achara Raksat,[a] Md Samiul Huq Atanu,[a] Karla J. McDermid,[b] Marisa Wall,[c] Boon Loong Chang,[d] Supakit Wongwiwatthananukit,[e] Leng Chee Chang[a,]*

[a]Department of Pharmaceutical Sciences, The Daniel K. Inouye College of Pharmacy, University of Hawai‘i at Hilo, Hilo, Hawaii, 96720, United States;

[b]Department of Marine Science, University of Hawai‘i at Hilo, 200 W. Kawili St., Hilo, Hawai‘i 96720, USA;

[c]Daniel K. Inouye U.S. Pacific Basin Agricultural Research Center, Hilo, Hawai‘i, 96720, United States;

[d]Department of Traditional Chinese Medicine, School of Pharmacy, Management and Science University, Shah Alam, Selangor, Malaysia;

[e]Department of Pharmacy Practice, The Daniel K. Inouye College of Pharmacy, University of Hawai'i at Hilo, Hilo, HI, 96720, USA

[*] **Corresponding Author to**: Tel: +1-808-981-8018, Fax: +1-808-933-2974.

E-mail address: lengchee@hawaii.edu

Table of Contents **Page**

**Figure S1**.	^{1}H NMR spectrum (400 MHz, MeOD) of compound **1**.	3
**Figure S2**.	^{13}C NMR spectrum (100 MHz, MeOD) of compound **1**.	3
**Figure S3**.	^{1}H NMR spectrum (400 MHz, MeOD) of compound **2**.	4
**Figure S4**.	^{13}C NMR spectrum (100 MHz, MeOD) of compound **2**.	4
**Figure S5**.	^{1}H NMR spectrum (400 MHz, MeOD) of compound **3**.	5
**Figure S6**.	^{1}H NMR spectrum (400 MHz, $CDCl_3$) of compound **4**.	5
**Figure S7**.	^{1}H NMR spectrum (400 MHz, $CDCl_3$) of compound **5**.	6
**Figure S8**.	^{13}C NMR spectrum (100 MHz, $CDCl_3$) of compound **5**.	6
**Figure S9**.	^{1}H NMR spectrum (400 MHz, $CDCl_3$) of compound **6**.	7
**Figure S10**.	^{13}C NMR spectrum (100 MHz, $CDCl_3$) of compound **6**.	7
**Figure S11**.	^{1}H NMR spectrum (400 MHz, $CDCl_3$) of compound **7**.	8
**Figure S12**.	^{13}C NMR spectrum (100 MHz, $CDCl_3$) of compound **7**.	8
**Figure S13**.	^{1}H NMR spectrum (400 MHz, $CDCl_3$) of compound **8**.	9
**Figure S14**.	^{1}H NMR spectrum (400 MHz, $CDCl_3$) of compound **9**.	9
**Figure S15**.	^{1}H NMR spectrum (400 MHz, $CDCl_3$) of compound **10**.	10
**Figure S16**.	^{13}C NMR spectrum (100 MHz, $CDCl_3$) of compound **10**.	10
**Figure S17**.	^{1}H NMR spectrum (400 MHz, $CDCl_3$) of compound **11**.	11

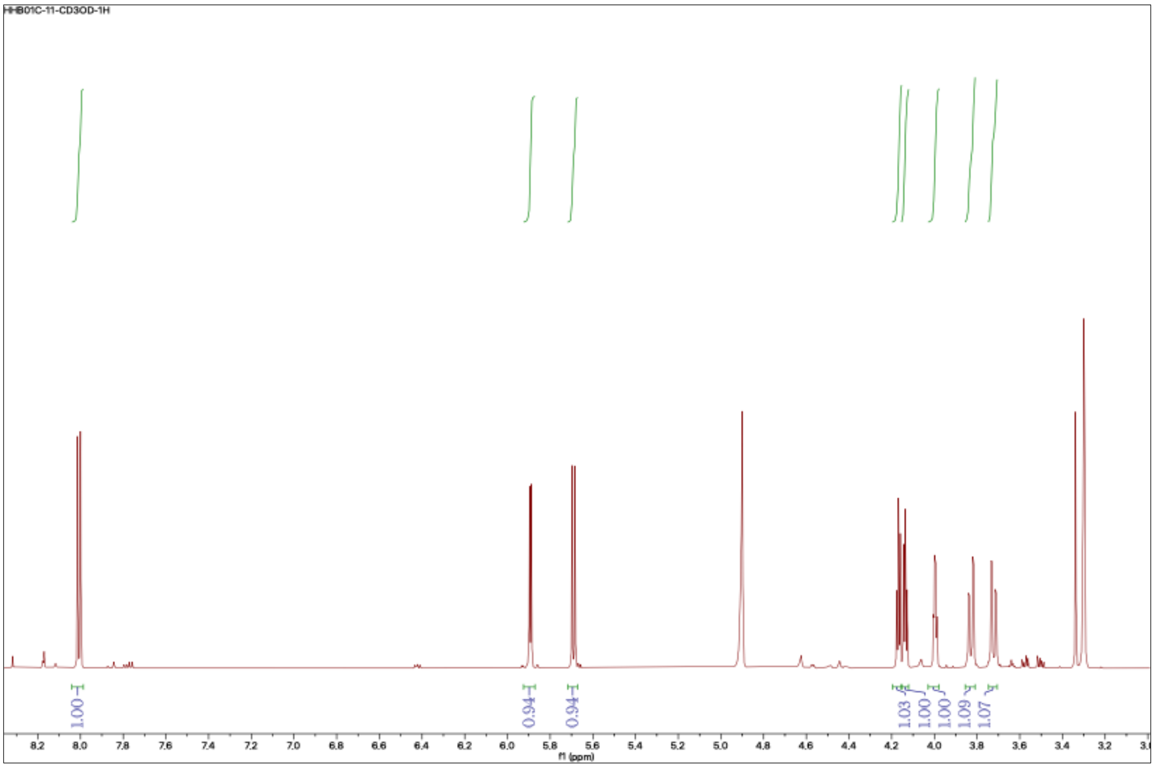

Figure S1. ^{1}H NMR spectrum (400 MHz, MeOD) of compound **1**.

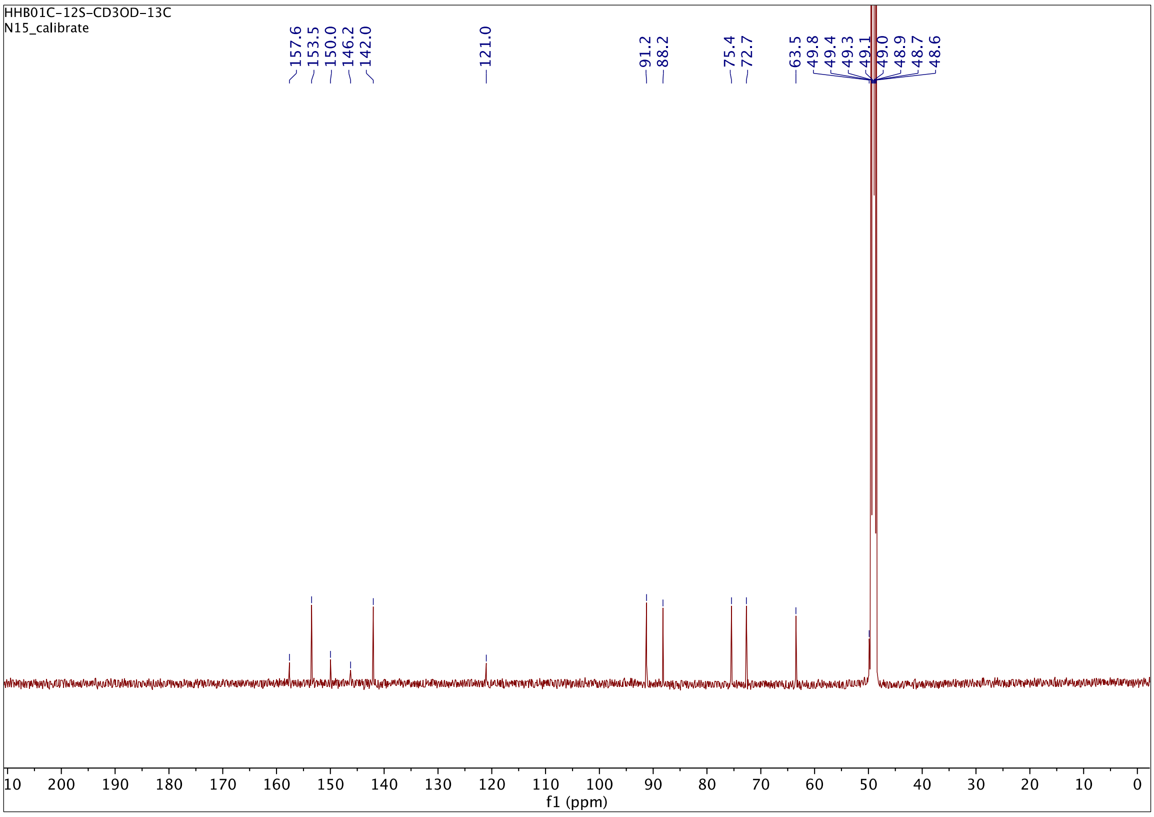

Figure S2. ^{13}C NMR spectrum (100 MHz, MeOD) of compound **1**.

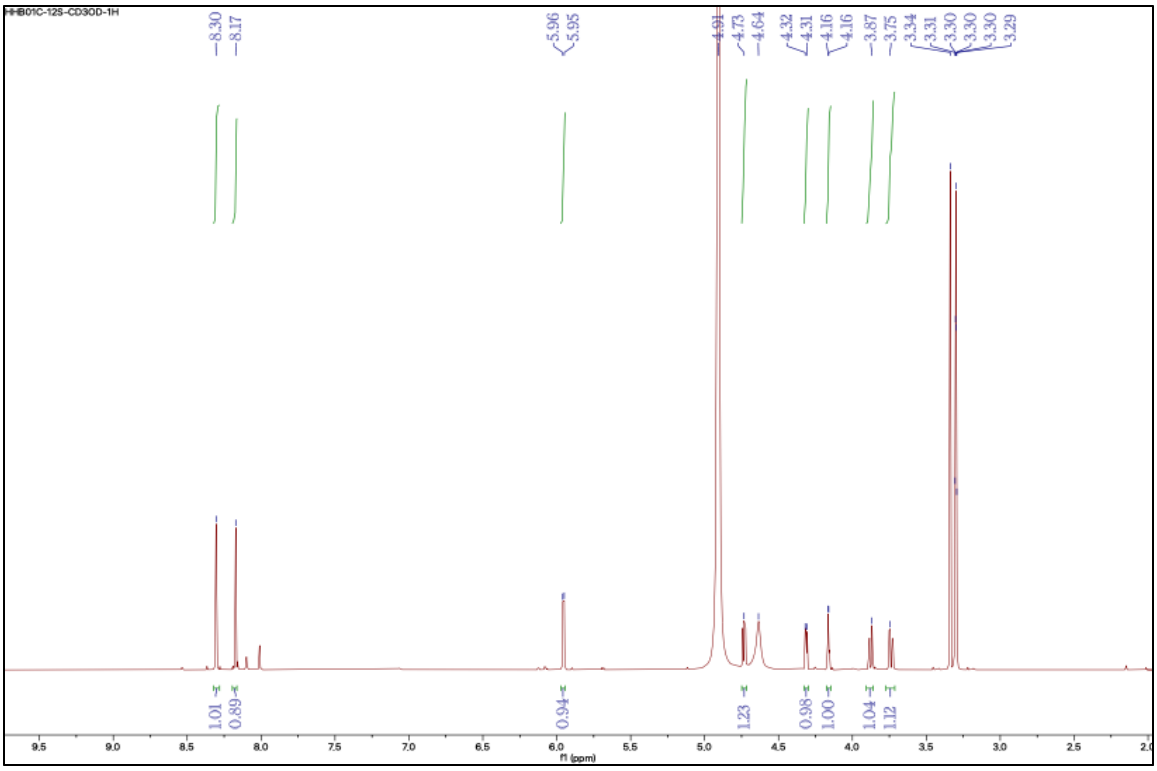

Figure S3. ^{1}H NMR spectrum (400 MHz, MeOD) of compound **2**.

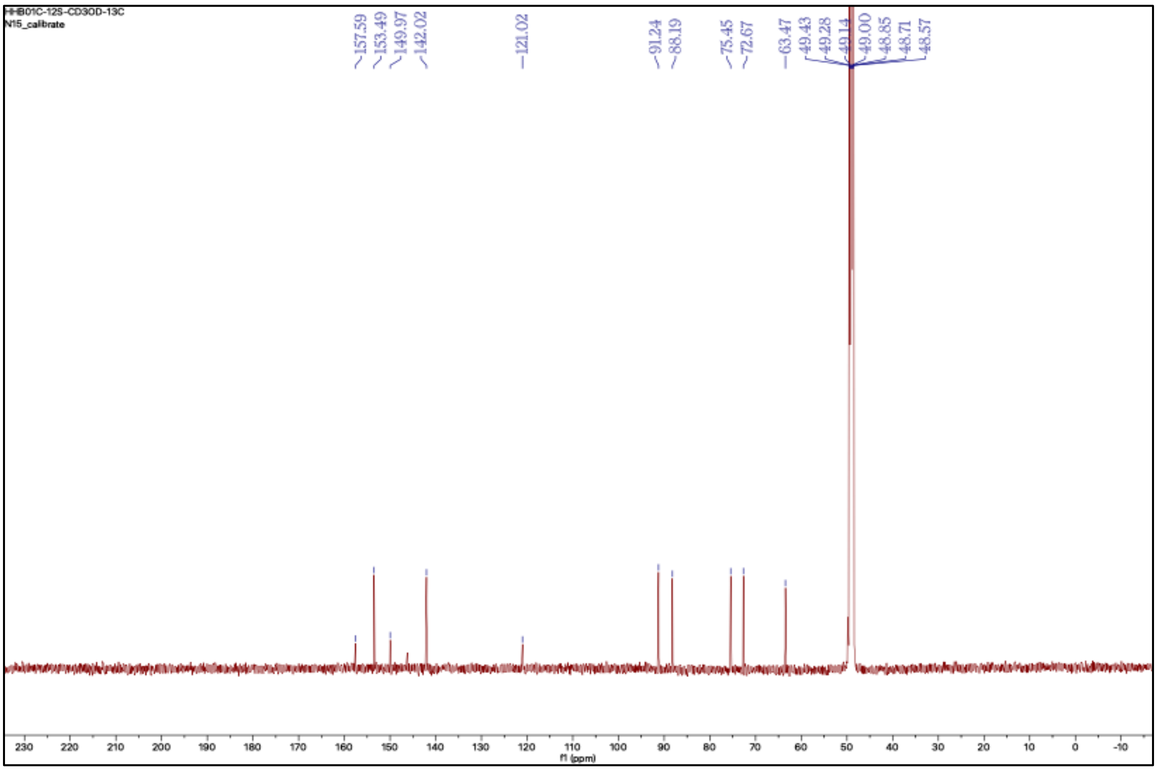

Figure S4. ^{13}C NMR spectrum (100 MHz, MeOD) of compound **2**.

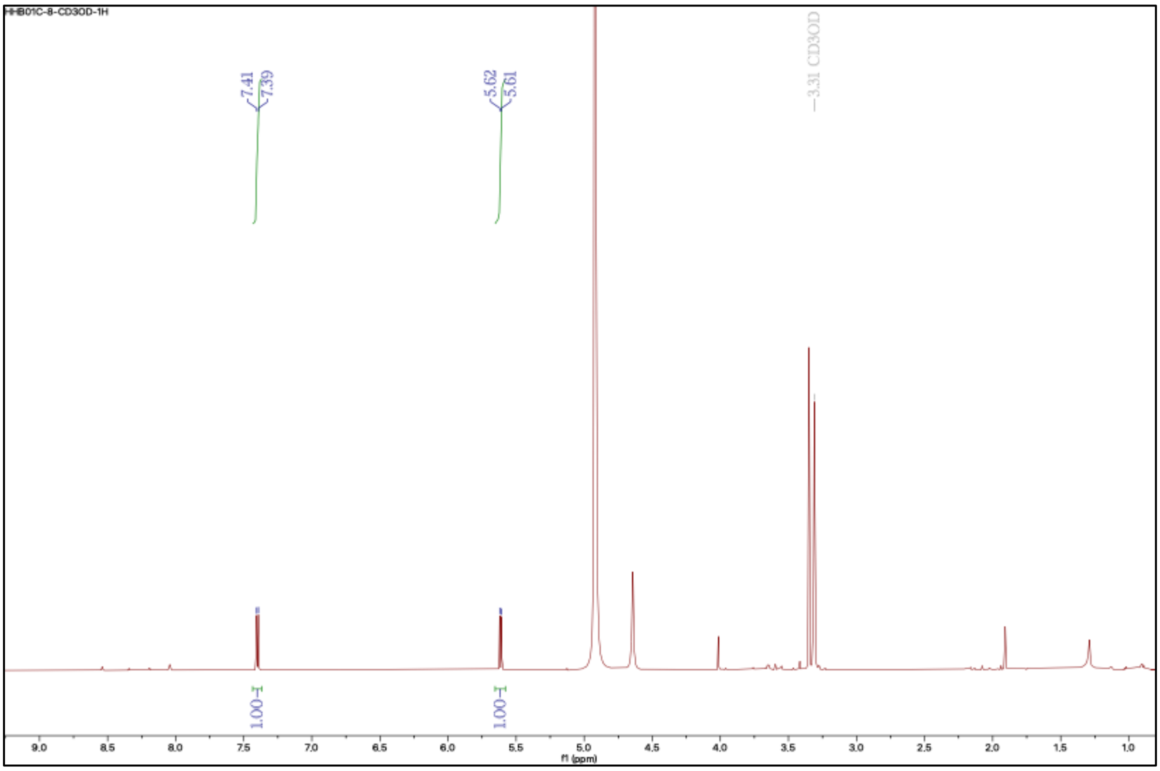

Figure S5. ^{1}H NMR spectrum (400 MHz, MeOD) of compound **3**.

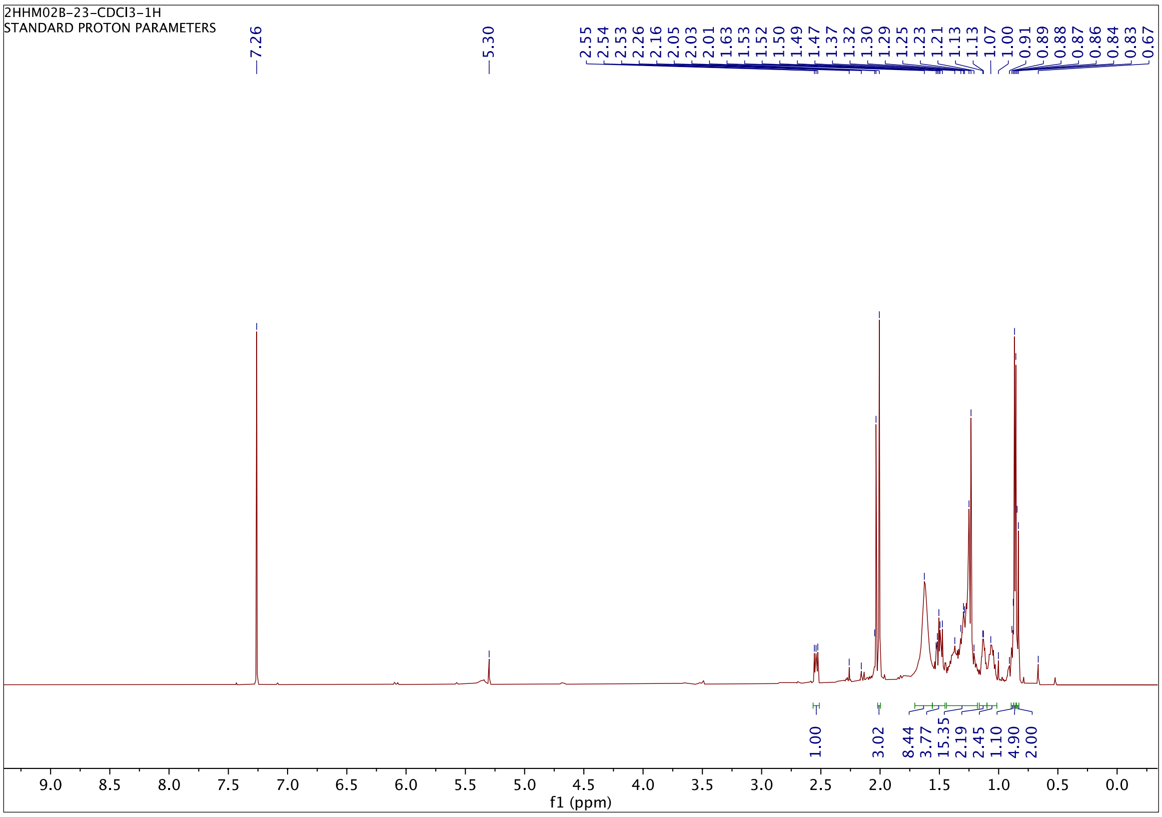

Figure S6. ^{1}H NMR spectrum (400 MHz, $CDCl_3$) of compound **4**.

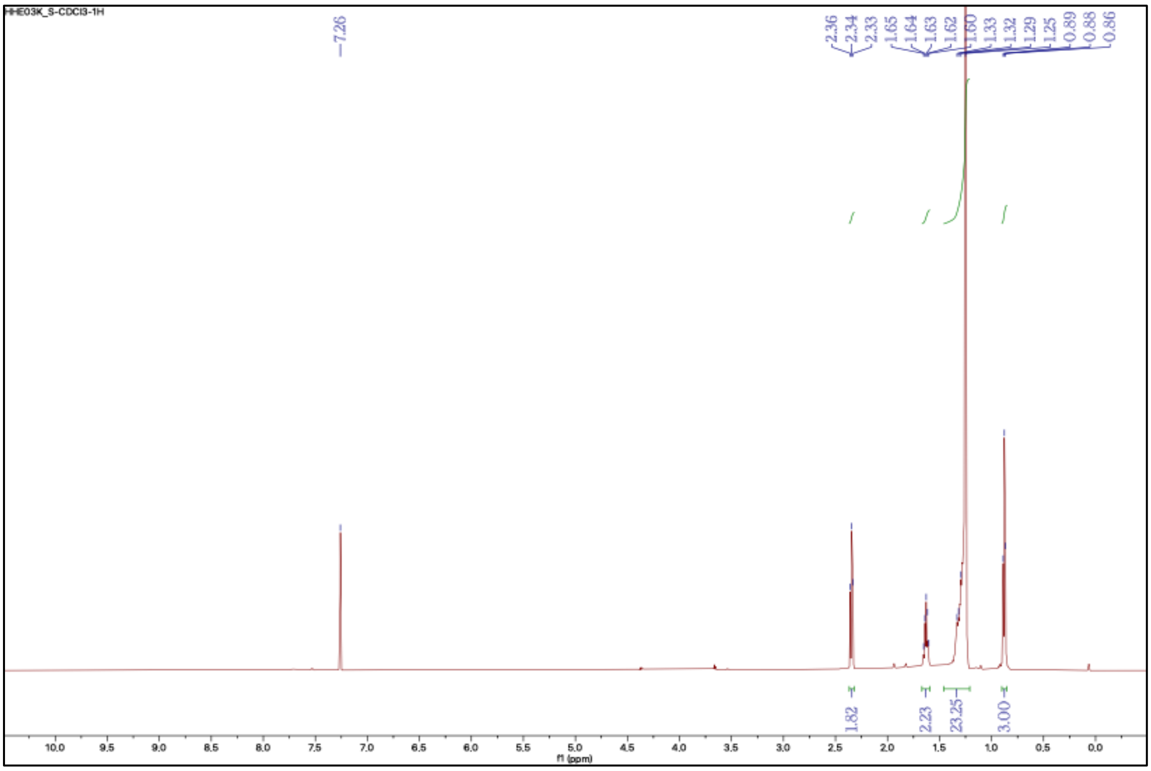

Figure S7. ^{1}H NMR spectrum (400 MHz, $CDCl_3$) of compound **5**.

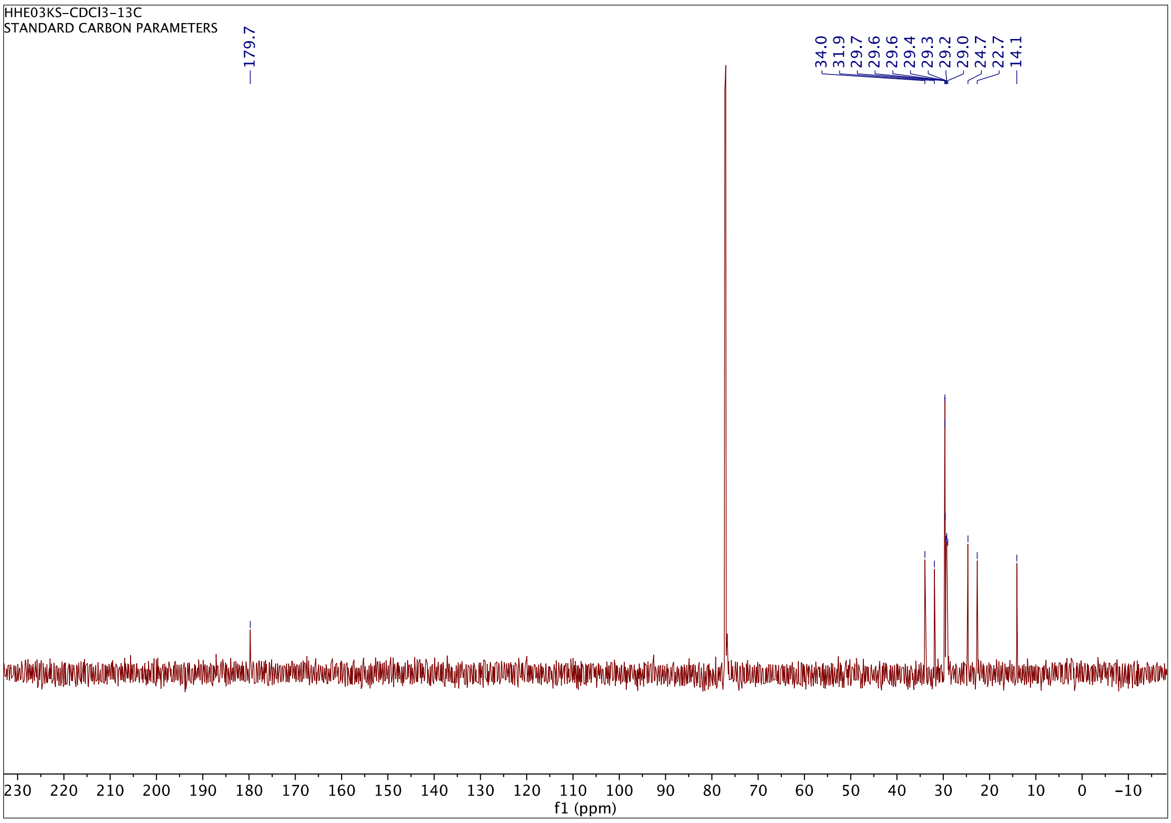

Figure S8. ^{13}C NMR spectrum (100 MHz, $CDCl_3$) of compound **5**.

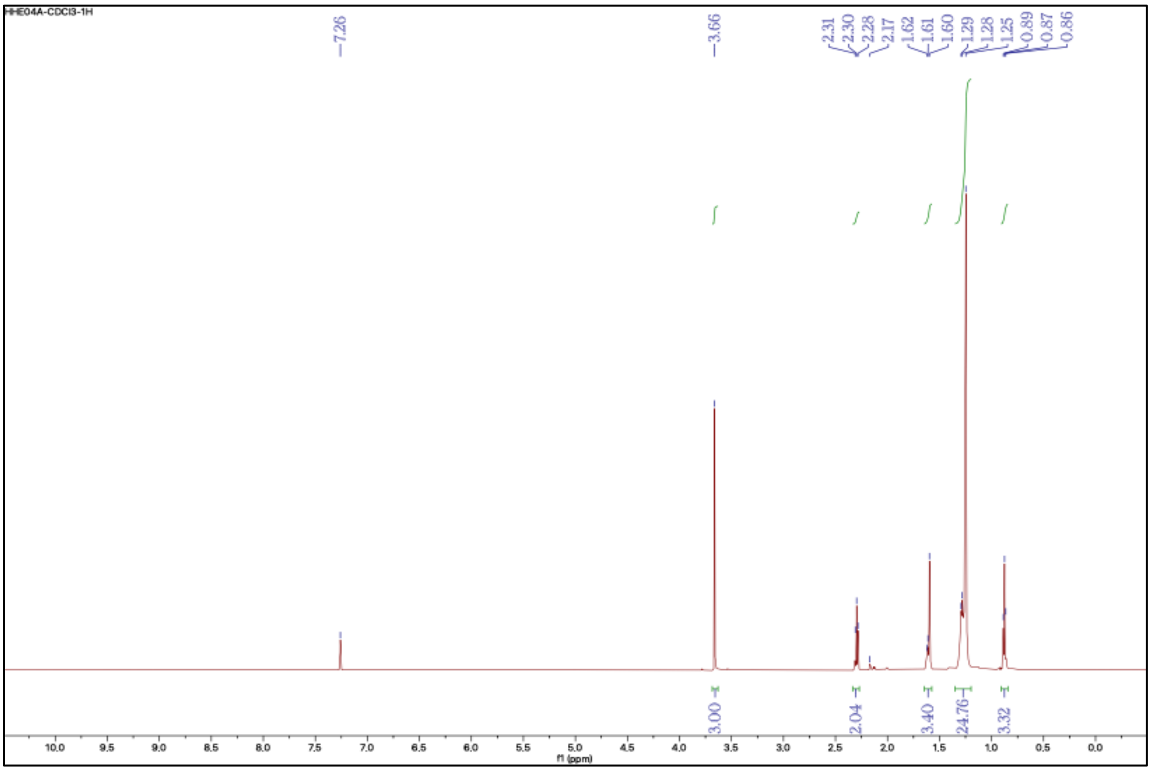

Figure S9. ^{1}H NMR spectrum (400 MHz, $CDCl_3$) of compound **6**.

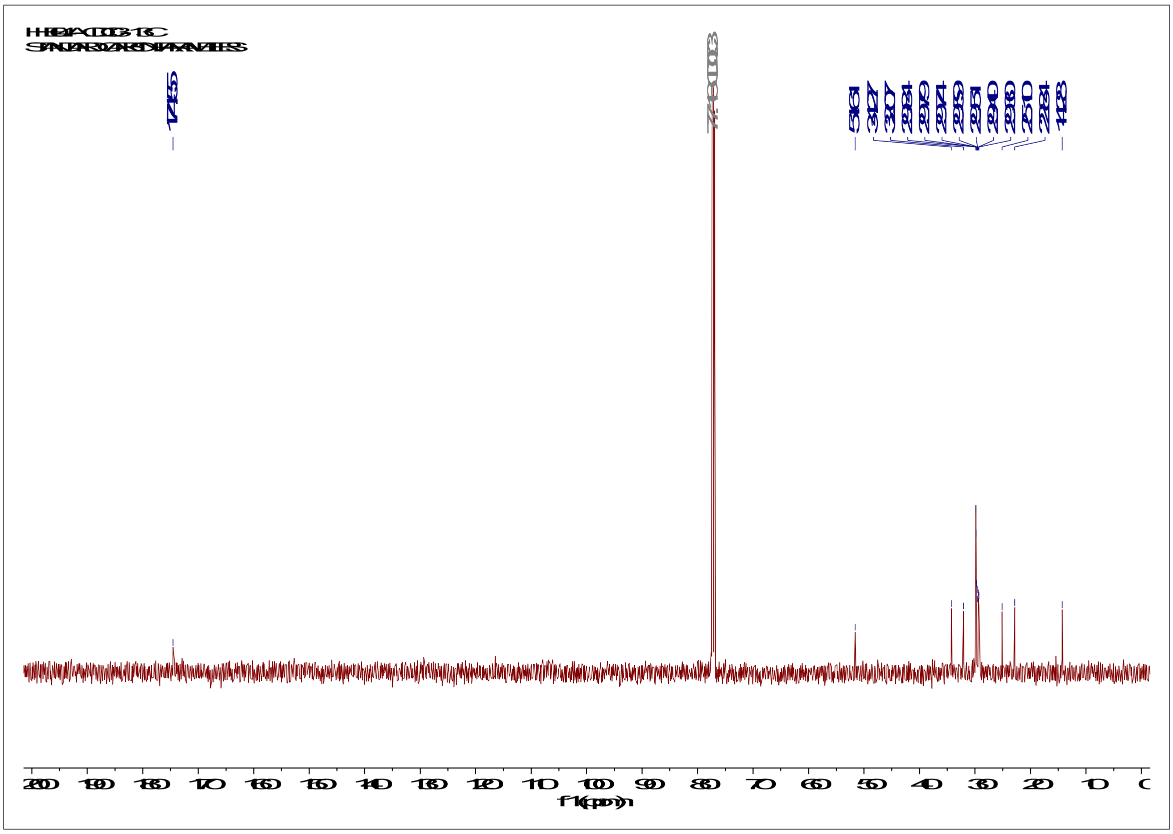

Figure S10. ^{13}C NMR spectrum (100 MHz, $CDCl_3$) of compound **6**.

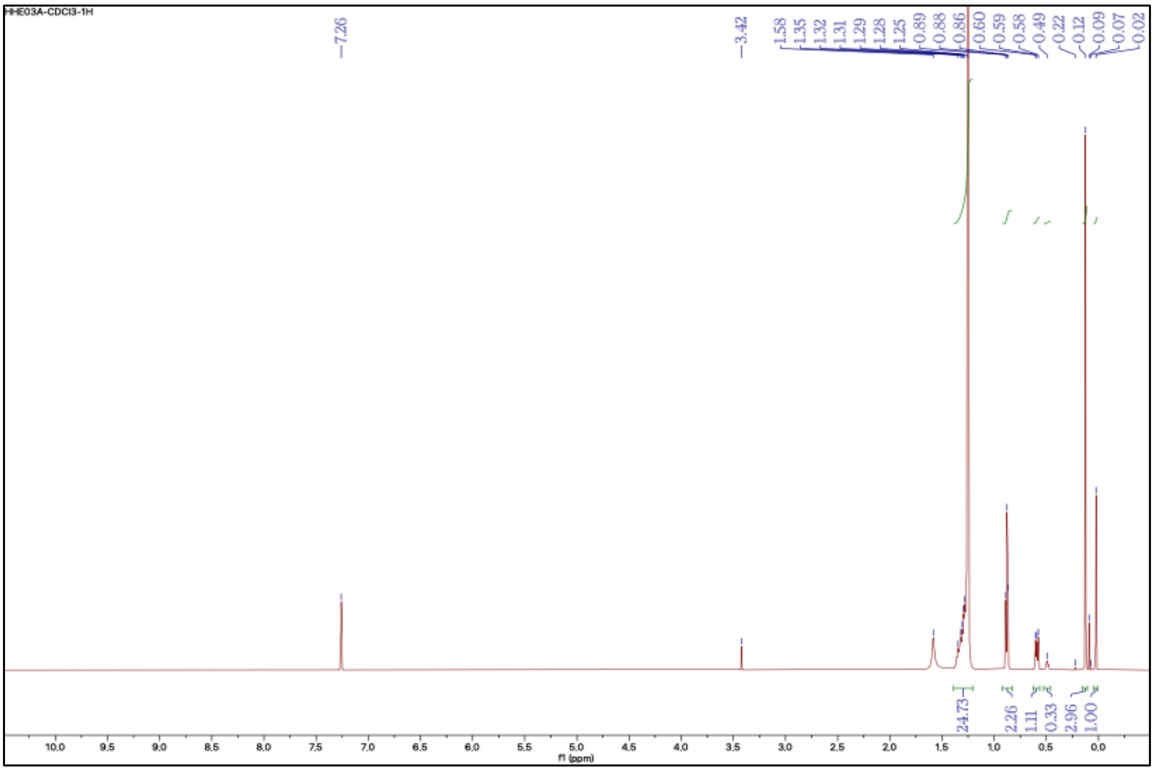

Figure S11. ^{1}H NMR spectrum (400 MHz, $CDCl_3$) of compound **7**.

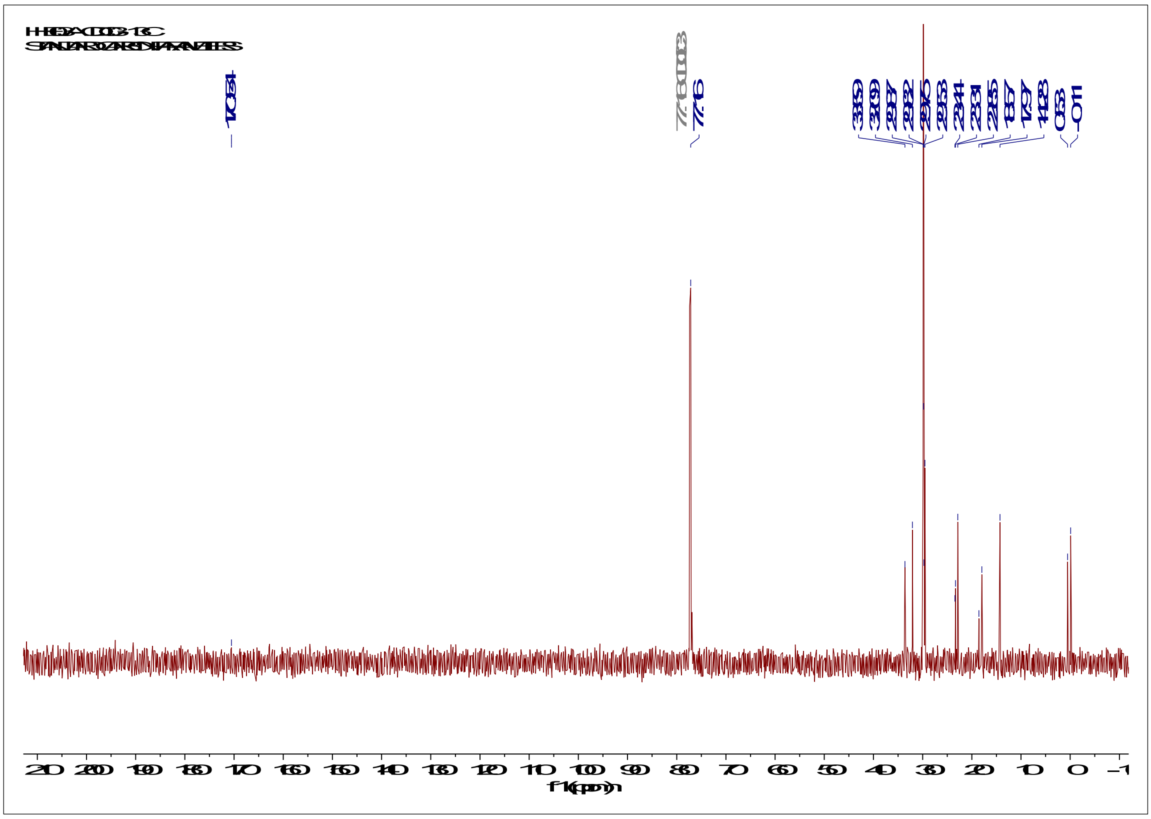

Figure S12. ^{13}C NMR spectrum (100 MHz, $CDCl_3$) of compound **7**.

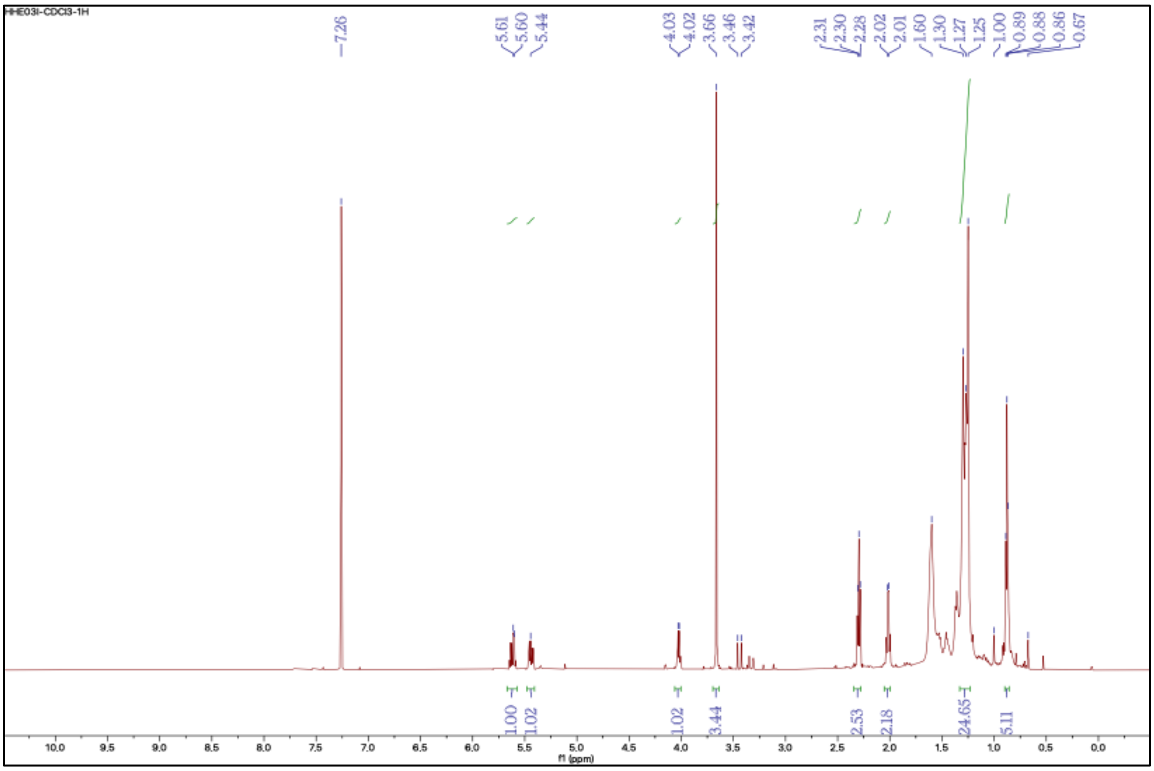

Figure S13. ^{1}H NMR spectrum (400 MHz, $CDCl_3$) of compound **8**.

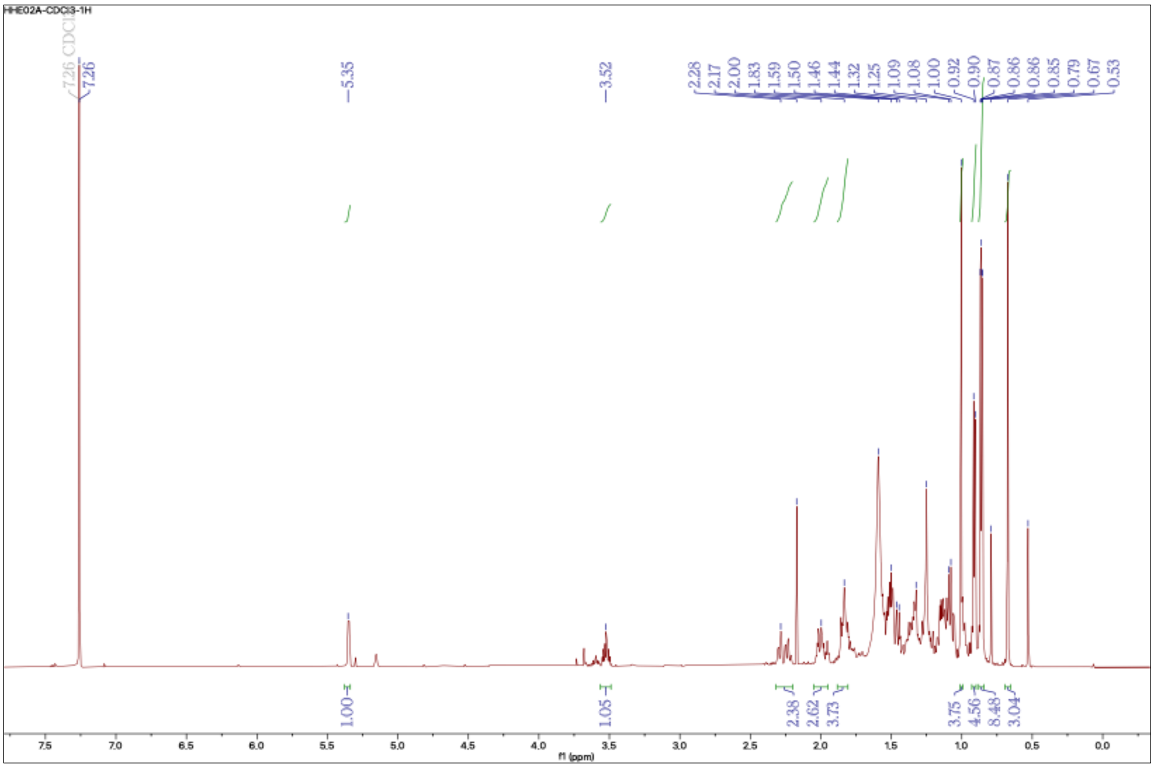

Figure S14. ^{1}H NMR spectrum (400 MHz, $CDCl_3$) of compound **9**.

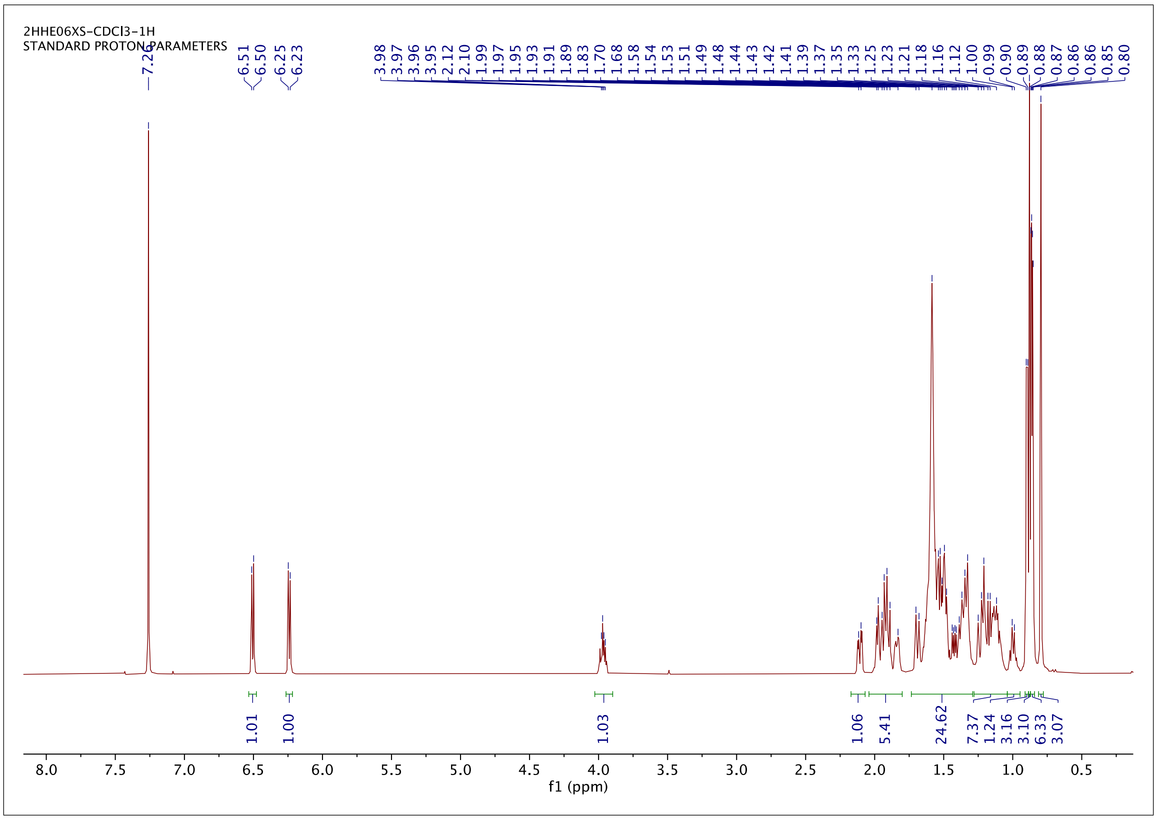

Figure S15. ^{1}H NMR spectrum (400 MHz, $CDCl_3$) of compound **10**.

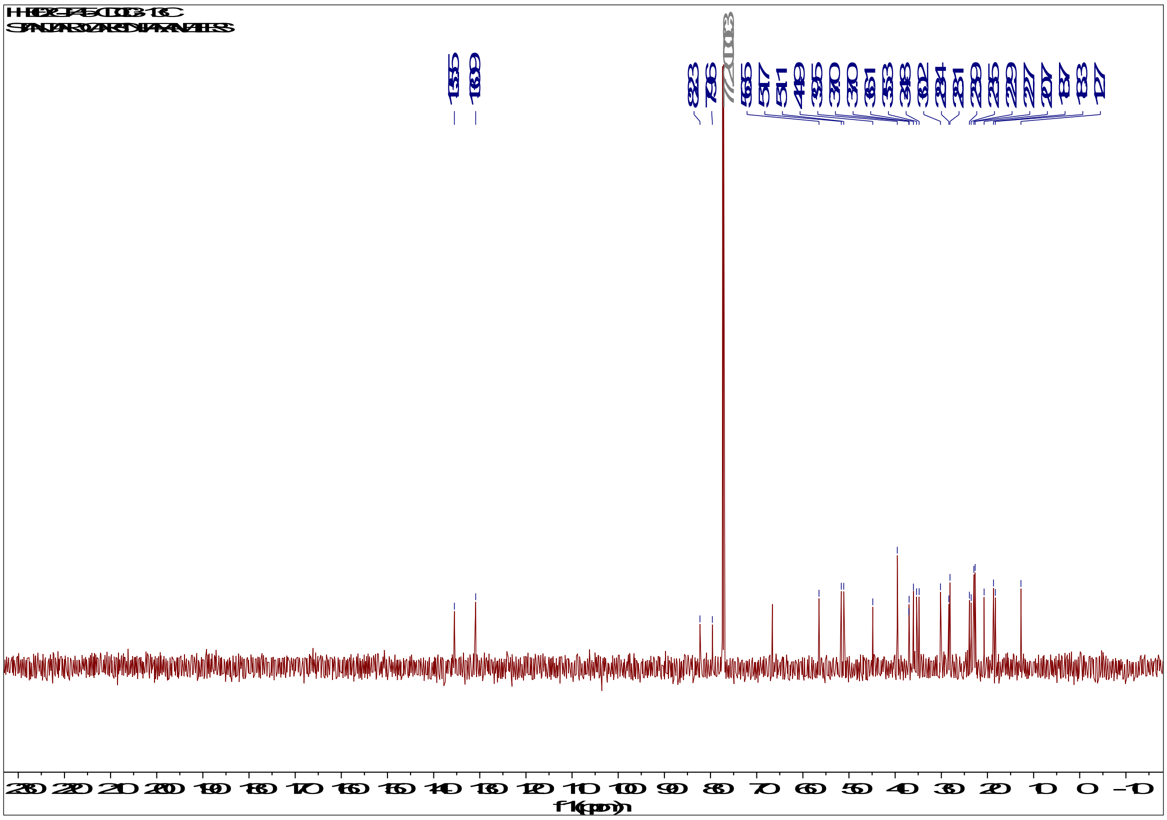

Figure S16. ^{13}C NMR spectrum (100 MHz, $CDCl_3$) of compound **10**.

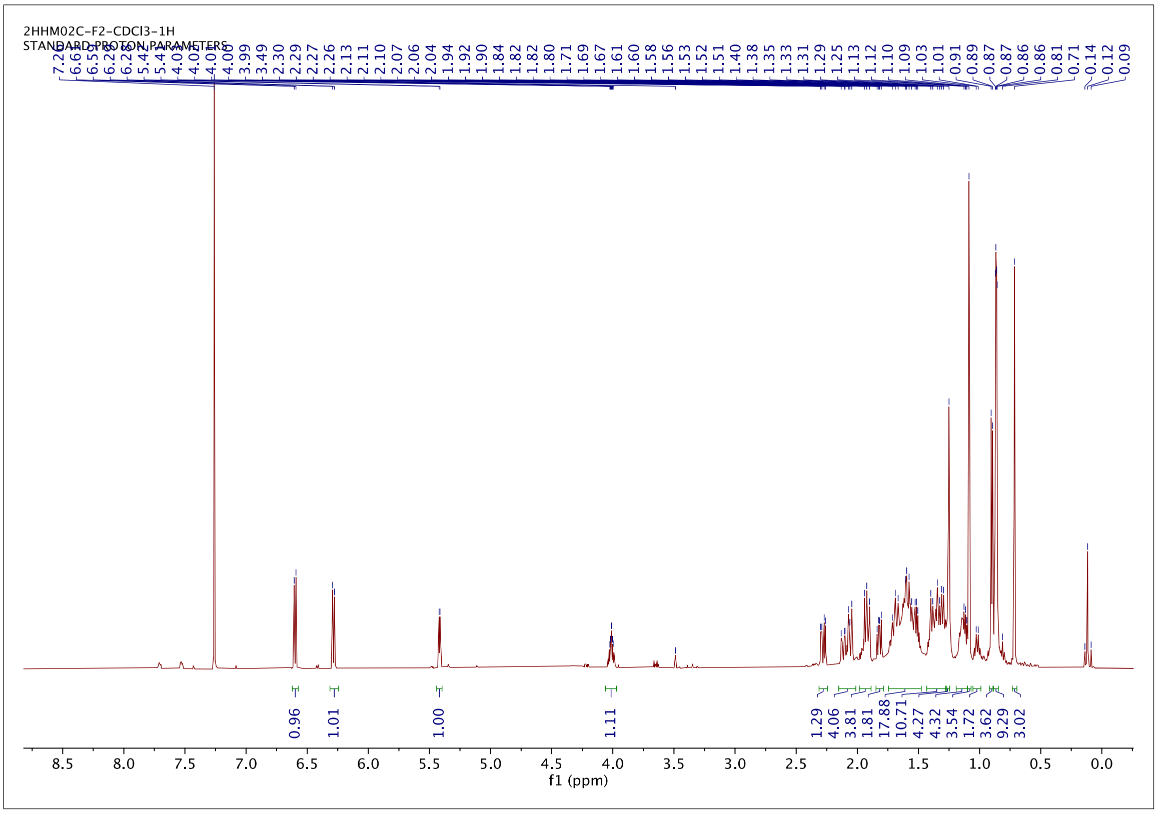

Figure S17. ^{1}H NMR spectrum (400 MHz, $CDCl_3$) of compound **11**.
